# Cross-talk of four types of RNA modification writers defines tumor microenvironment and pharmacogenomic landscape in colorectal cancer

**DOI:** 10.1186/s12943-021-01322-w

**Published:** 2021-02-08

**Authors:** Huifang Chen, Jiameng Yao, Rujuan Bao, Yu Dong, Ting Zhang, Yanhua Du, Gaoyang Wang, Duan Ni, Zhenzhen Xun, Xiaoyin Niu, Youqiong Ye, Hua-Bing Li

**Affiliations:** 1 Shanghai Institute of Immunology, Department of Immunology and Microbiology, Shanghai Jiao Tong University School of Medicine, Shanghai, 200025 China; 2 The Charles Perkins Centre, University of Sydney, Sydney, NSW 2006 Australia; 3 Shanghai Jiao Tong University School of Medicine-Yale Institute for Immune Metabolism, Shanghai Jiao Tong University School of Medicine, Shanghai, 200025 China; 4 Department of Liver Surgery, State Key Laboratory of Oncogenes and Related Genes, Renji Hospital, Shanghai Jiao Tong University School of Medicine, Shanghai, 200127 China

**Keywords:** RNA modification “writer”, Colorectal cancer, Tumor microenvironment, WM_Score, Drug sensitivity, Immunotherapy

## Abstract

**Background:**

The four major RNA adenosine modifications, i.e., m^6^A, m^1^A, alternative polyadenylation, and adenosine-to-inosine RNA editing, are mediated mostly by the “writer” enzymes and constitute critical mechanisms of epigenetic regulation in immune response and tumorigenesis. However, the cross-talk and potential roles of these “writers” in the tumor microenvironment (TME), drug sensitivity, and immunotherapy remain unknown.

**Methods:**

We systematically characterized mRNA expression and genetic alterations of 26 RNA modification “writers” in colorectal cancer (CRC), and evaluated their expression pattern in 1697 CRC samples from 8 datasets. We used an unsupervised clustering method to assign the samples into two patterns of expression of RNA modification “writers”. Subsequently, we constructed the RNA modification “writer” Score (WM_Score) model based on differentially expressed genes (DEGs) responsible for the RNA modification patterns to quantify the RNA modification-related subtypes of individual tumors. Furthermore, we performed association analysis for WM_Score and characteristics of TME, consensus molecular subtypes (CMSs), clinical features, transcriptional and post-transcriptional regulation, drug response, and the efficacy of immunotherapy.

**Results:**

We demonstrated that multi-layer alterations of RNA modification “writer” are associated with patient survival and TME cell-infiltrating characteristics. We identified two distinct RNA modification patterns, characterized by a high and a low WM_Score. The WM_Score-high group was associated with worse patient overall survival and with the infiltration of inhibitory immune cells, such as M2 macrophages, EMT activation, and metastasis, while the WM_Score-low group was associated with a survival advantage, apoptosis, and cell cycle signaling pathways. WM_Score correlated highly with the regulation of transcription and post-transcriptional events contributing to the development of CRC. ﻿In response to anti-cancer drugs, WM_Score highly negatively correlated (drug sensitive) with drugs which targeted oncogenic related pathways, such as MAPK, EGFR, and mTOR signaling pathways, positively correlated (drug resistance) with drugs which targeted in apoptosis and cell cycle. Importantly, the WM_Score was associated with the therapeutic efficacy of PD-L1 blockade, suggesting that the development of potential drugs targeting these “writers” to aid the clinical benefits of immunotherapy.

**Conclusions:**

Our study is the first to ﻿provide a comprehensive analysis of four RNA modifications in CRC. We revealed the potential function of these writers in TME, transcriptional and post-transcriptional events, and identified their therapeutic liability in targeted therapy and immunotherapy. This work highlights the cross-talk and potential clinical utility of RNA modification “writers” in cancer therapy.

**Supplementary Information:**

The online version contains supplementary material available at 10.1186/s12943-021-01322-w.

## Introduction

Colorectal cancer (CRC) is the third most prevalent cancer and the second most frequent cause of cancer-related deaths worldwide [1]. Previous studies have suggested that the onset of CRC results from the accumulation of mutations in genes controlling key signaling pathways, such as RAS-MAPK, WNT, and PI3K [2]. While somatic mutations may be partly responsible for the development of CRC, epigenetic changes in cancer-related genes and genes regulating inflammatory responses are also implicated in the etiology of CRC [3]. Epigenetics is a branch of genetics that studies stable and heritable phenotypes caused by chromosomal changes that do not alter the nucleotide sequence of genes [4]. Recently, an increasing number of studies have shown that RNA modification is an important mechanism of epigenetic regulation and plays an important role in the physiological process of the organism as well as the in occurrence and development of diseases [3].

In nature, RNA modification is widespread on all nucleotides: A, U, C, and G [5]. There are over 170 modifications in RNA levels, including m^5^C, m^3^C, m^7^G, Pseudouracil(ψ), Nm modification [6–10]. It is possible that many of those modifications could interact, but so far is impossible to include all of them in the study. Since Adenine is the nucleotide on RNA that is most heavily modified, and modification on one of nitrogen atom of the Adenine base, such as m^1^A, carries a positive charge under physiological conditions [11], it is likely that there is some competitively compensated interaction between the modifications. One report already showed that m6A modification “writer” could negatively regulate the A-to-I modification [12]. Therefore, we focused on adenine-related RNA modification including m^6^A methylation, m^1^A methylation, APA, and A-to-I RNA editing. These modifications are mainly produced by the activity of enzymes known as “writers”.

m^6^A is methylation at the sixth nitrogen atom of RNA base A. It is the most abundant form of internal RNA modifications, affecting RNA stability and translational efficiency. This modification is written by m^6^A-methyltransferases, such as METTL3, METTL14, WTAP, RBM15, RBM15B, ZC3H13, and KIAA1429 [13]. The presence of m^6^A can cause profound changes in cellular processes and plays a key role in pathological conditions, including the development of cancer [14–17].

The modification of m^1^A affects the first nitrogen atom of the adenine base and carries a positive charge under physiological conditions [18]. Known m^1^A modification “writers” include TRMT61A, TRMT61B, TRMT10C, and TRMT6 [19, 20]. m^1^A modification affects the tertiary structure of ribosomes and the translation of genes. It has an essential function in regulating gene expression and controlling cell fate, thus affecting the occurrence and progression of diseases [21, 22].

APA is an RNA-processing mechanism that cleaves mRNA at different sites and adds poly(A) tails to generate transcripts containing different lengths of 3′-untranslated region (UTR) or coding regions [23, 24]. CPSF, CSTF, CFI, PCF11, CLP1, NUDT21, and PABPN1 protein complex can regulate poly(A) site selection, shear, and poly(A) tail synthesis [24, 25]. APA mediated by CFI is linked to the suppression of glioblastoma. In highly proliferating cells, the proximal poly(A) site is extensively used to form mRNA with a shorter 3’UTR [26].

RNA editing is a well-documented post-transcriptional mechanism altering nucleotide in selected transcripts [27]. The common type of RNA editing is A-to-I, which is catalyzed by ADAR enzymes, including ADAR, ADARB1, and ADARB2. The A-to-I editing can change the sequence of amino acids in the protein and affect other transcription processes, thereby contributing to tumorigenesis and tumor progression by site-specific modifications of tumor-related genes [28, 29].

To fully understand the significance of post-transcriptional modifications, the investigation of cross-talk between different patterns of these alterations is urgently needed. The four types of RNA modification “writers” may form an important and complex cellular regulatory network in CRC, and the understanding of this network may provide important insights into the mechanisms underlying CRC tumorigenesis.

The immune checkpoint blockade (ICB) therapy has been applied for cancer treatment and delivered promising clinical outcomes; however, it generally shows a low response rate. To improve the efficacy of immunotherapy, dissecting the tumor microenvironment (TME) and identifying the mechanism underlying the low rate of response rate to ICB are urgently needed [30]. Recent studies have shown that mRNA modification and related enzymes are highly associated with the microenvironment of tumors and immune cells. METTL3-mediated m^6^A modification promotes the activation and maturation of dendritic cells (DCs). Specific depletion of Mettl3 in DCs resulted in an impaired phenotypic and functional maturation of DCs and reduced their ability to stimulate T cell responses [31]. Distinct patterns of 3’UTR have been detected across different immune cells or other cell types present in TME [32]. However, due to the limitations in methodology, these studies have been confined to only one or two RNA modification “writers”, while the antitumor effect of RNA modification is characterized by highly integrated interaction of numerous regulators. Therefore, a comprehensive understanding of how the regulatory network of multiple RNA modification “writers” affects the TME cells will contribute to our understanding of the immune regulation in the TME and the development of immunotherapeutic strategies.

In this study, we explored genomic alterations in 1697 CRC samples from Gene Expression Omnibus (GEO) [33–40] and The Cancer Genome Atlas (TCGA) [41] cohort and evaluated the patterns of RNA modifications. We found that RNA modification patterns were not only associated with the infiltration of multiple immune cell types, but also with the activation of epithelial-mesenchymal transition (EMT), cell cycle, and apoptosis. Next, based on differentially expressed genes (DEGs) in the RNA modification patterns, we developed the “writers” of RNA modification score (WM_Score) model to quantify the efficacy of “writers” in individual patients. Finally, we demonstrated the applicability of the WM_Score to distinguish the transcriptional and post-transcriptional events, and assessed its therapeutic value in targeted therapy and immunotherapy.

## Results

### Genetic and transcriptional alterations of four types of RNA modification “writers” in CRC

Based on the published data, a total of 26 RNA modification “writers” (Table S1), including 3 A-I modification “writers”, 7 m^6^A modification “writers”, 4 m^1^A modification “writers”, and 12 APA modification “writers” were included in the current study [13, 19, 24, 28].

**Fig. 1:**
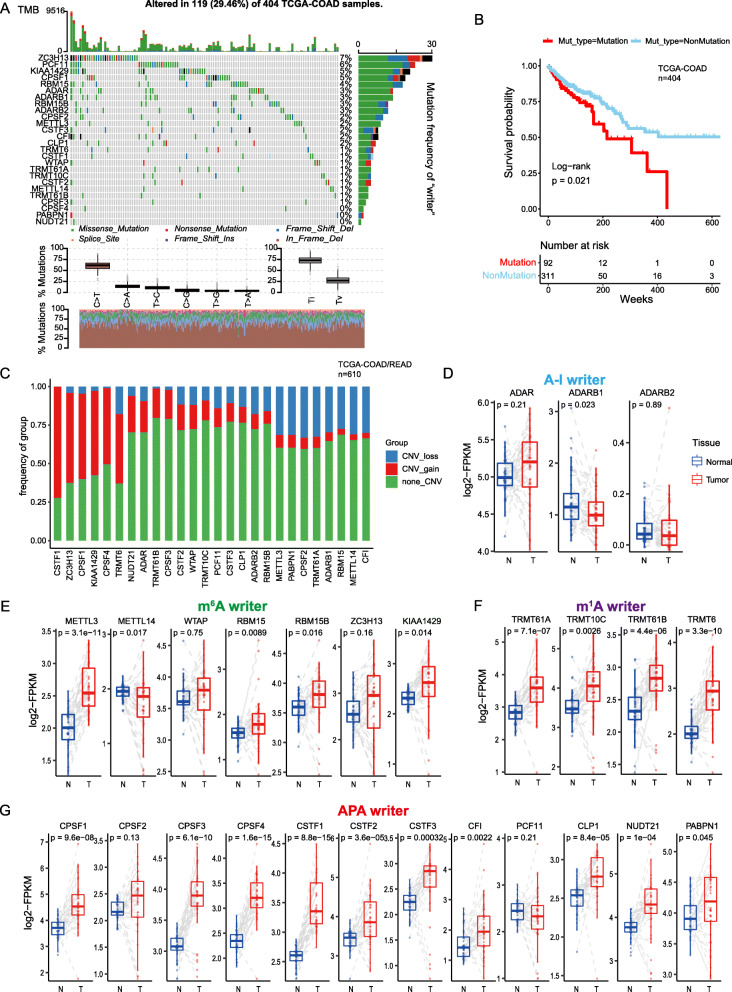
Genetic and transcriptional alterations of RNA modification “writers” in CRC. **a** The mutation frequency of 26 RNA modification “writers” in 404 CRC patients from the TCGA cohort. Each column represents individual patients. The upper bar graph shows TMB; the number on the right indicates the mutation frequency in each “writer”. The right bar graph shows the proportion of each variant type. The stacked bar graph below shows the fraction of conversions in each sample. **b** Kaplan-Meier curves show overall survival of patients with (red) or without (blue) mutations in RNA modification “writer” in the COAD cohort. The grouping of CRC samples is indicated at the bottom of the chart. *p* < 0.05 in the two-sided log-rank test was considered statistically significant. **c** Bar graphs showing the frequency of CNV gain (red), loss (blue) and non_CNV (green) of RNA modification “writers” in the TCGA-COAD/READ cohort. The height of each bar represents the alteration frequency. **d-g** Box plots show the expression distribution of 26 “writers” of 4 types RNA modification between paired normal (blue) and CRC (red) tissues; the paired samples are connected by gray dash lines. The boxes indicate the median ± 1 quartile, with the whiskers extending from the hinge to the smallest or largest value within 1.5× IQR from the box boundaries

To determine the genetic alterations in RNA modification writers in cancer, we assessed the prevalence of non-silent somatic mutations in 26 writers. The mutation frequency of individual writers was relatively low across LAML, PCPG, and UVM cohorts in TCGA, while the COAD cohort showed relatively high mutation frequency of “writers” (Figure S2A). Of the 404 COAD samples, 119 (29.46%) had mutations of RNA modification “writers” (Fig. 1a). Among them, the mutation frequency of ZC3H13 was the highest (7%), followed by PCF11 and KIAA1429, while PABPN1 and NUDT21 did not show any mutations in CRC samples. However, CRC patients with mutations of these “writers” had shorter overall survival than those without mutations (Fig. 1b; log-rank test, *p* = 0.021), suggested that genetic alteration of “writer” may play functional role in CRC. Next we performed Gene Set Variation Analysis (GSVA) of enrichment analysis [42] using the hallmark gene sets to compare the “writers’“mutation group with the non-mutation group. We found that in the mutation group, more cancer hallmarks related gene sets were enriched, such as KRAS signaling pathway, TNFα signaling pathway, P53 signaling pathway, and IL6/JAK/STAT3 signaling pathway (Figure S2B). IL-6 is a pleiotropic cytokine in immune and inflammatory responses and plays an important role through the JAK3/STAT3/SOCS3 pathway. Moreover, IL-6-mediated dysregulation of the JAK/STAT3/SOCS3 signaling pathway is closely related to the formation of CRC [43, 44]. This suggests that the mutation of “writer” is likely to lead to functional changes that affect the survival prognosis of CRC.

We then examined ﻿﻿somatic copy number alterations of these “writers” and found that CSTF1, CPSF1/4, ZC3H13, and KIAA1429 had a widespread frequency of copy number variation (CNV) gain (Fig. 1c). To ascertain whether these genetic variations impacted the expression of RNA modification “writers” in CRC patients, we compared the mRNA alterations of regulators between paired normal and CRC samples and showed that the expression of a majority of the “writers” was significantly increased in CRC (Fig. 1d-g). Compared to the normal colon tissue, RNA modification “writers” with CNV gain (e.g., CSTF1 and CPSF1) were markedly more frequent in CRC tissues (Fig. 1c and g), suggested CNV may be a regulator factor to mRNA expression of “writer”. However, some “writer” showed upregulated mRNA expression but with high frequency of CNV loss. To investigate the discrepancy between CNV values and mRNA expression in tumor sample, we focused on eight writers which showed CNV loss in more than 20% of the samples, and divided the CRC cohort into four groups based on their CNV values, including CNV gain, CNV loss and or non-significant alteration of CNV. Then, we compared mRNA expression of “writer” between these groups (Figure S2C). Indeed, patients with CNV gain showed higher expression than those with CNV loss in these “writers”. METTL14, ADORB1, CPSF2, and PABPN1 showed significant downregulation or non-significant alteration in CNV loss group compared to normal tissues. Tumorigenesis is complex process, CNV changes could not fully explain the differential the expression of “writers” between tumor and normal tissues. Although many of the detected expression alterations of “writers” may be explained by CNVs, the CNV is a partial but not unique factor to regulate mRNA expression [45]. Other features, including DNA methylation and transcription factors, can regulate gene expression [46–48].

This analysis demonstrated a high heterogeneity of genetic landscape and expression of RNA modification “writers” between normal and CRC samples, indicating that the expression imbalance of RNA modification “writers” has potential roles in the onset and development of CRC.

### Distinct patterns of RNA modification “writers” associated with cancer hallmarks and immune infiltration

To gain a comprehensive understanding of the expression pattern of the “writers” involved in tumorigenesis, 1695 CRC samples from eight datasets that contained clinical information (GSE41568, GSE39582, GSE13294, GSE14333, GSE18105, GSE20916, GSE21510, GSE37892) were selected for further analysis (Table S2). Univariate Cox regression showed that 10 of 26 RNA modification “writers’“correlated with CRC prognosis in the GSE39582 dataset (Figure S3A).

**Fig. 2:**
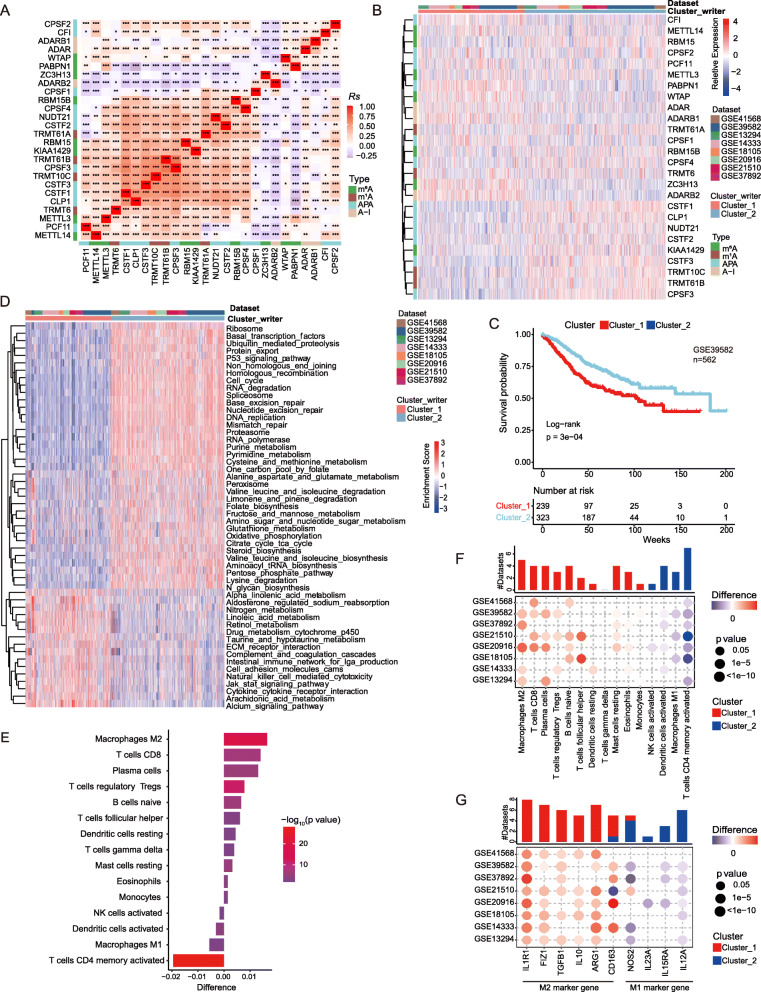
Patterns of RNA modification and biological characteristics of each pattern. **a** Heatmap shows a positive (red) and negative (blue) correlation among RNA modification “writers” in CRC. *p < 0.05, **p < 0.01, and ***p < 0.001, as determined by the Spearman correlation analysis. **b** Unsupervised clustering of 26 RNA modification “writers”. The clusters of CRC cohorts and RNA modification type were used as sample annotations. Red, high expression of “writers”; blue, low expression. **c** A heatmap visualizing the GSVA enrichment analysis shows the activation states of biological pathways in distinct RNA modification patterns. Red, activated pathways; blue, inhibited pathways. The names of CRC cohorts were used as sample annotations. **d** Kaplan-Meier curves compare overall survival between two RNA modification patterns, Cluster_1 (red) and Cluster_2 (blue), in GSE39582. The grouping of CRC samples is shown at the bottom of the chart. *p* < 0.05 in the two-sided log-rank test was considered statistically significant. **e** The difference in the relative abundance of immune cell infiltration in TME between RNA modification Cluster_1 and RNA modification Cluster_2 was calculated by the CIBERSORT algorithm. Difference > 0 indicates that the immune cells were enriched in RNA modification Cluster_1, and the column color represents the statistical significance of the difference. **f-g** The difference of immune cell infiltration (**f**) and expression of macrophage marker genes (**g**) between RNA modification patterns. The upper bar graph shows the number of datasets that differ significantly between Cluster_1 and Cluster_2. The color of the bubble below the graph indicates the difference in each of the distinct GEO datasets, and the bubble size indicates the statistical significance of the difference. Difference > 0 indicates that the infiltration of immune cells (**f**) or expression of macrophage marker genes (**g**) were higher in RNA modification Cluster_1

To explore the relationship among writers, we calculated pairwise correlations among the expression of 26 writers in CRC and found that positive correlations were more frequent than negative correlations (Fig. 2a). We identified that not only the expression of RNA modification “writers” was remarkably correlated in the same category, but also a significant correlation was present among different types of modification writers. Notably, the expression of ADARB2, ZC3H13, and PABPN1 was negatively correlated with other “writers”. While the expressions of TRMT61B, TRMT6, KIAA1429, TRMT10C, CSTF2/3, CPSF4, and CLP1 were positively correlated to each other (Fig. 2a). Thus, cross-talk among the “writers” may be important for the generation of different RNA modification patterns between individual tumors.

Next, we applied Consensus Clustering based on the expression profiles of 26 selected RNA modification “writers” to classify patients with qualitatively different RNA modification patterns (Table S3). After unsupervised clustering, 727 CRC patients from the combined datasets were identified in Cluster_1, whereas the other 968 patients were identified in Cluster_2 (Fig. 2b). In the prognostic analysis of RNA modification patterns, subtypes revealed a particularly prominent survival advantage in the Cluster_2 modification pattern (Fig. 2c; log-rank test, *p* = 3.0 × 10^− 5^). To identify the biological significance of these distinct RNA modification patterns, we performed GSVA enrichment analysis (Table S5). Cluster_1 was markedly enriched in stromal and carcinogenic activation pathways such as the ECM receptor interaction, TGF-β signaling pathway, and cell adhesion, suggesting that RNA modification “writers” may be associated with tumorigenesis. Cluster_2 was enriched in pathways associated with proliferation and apoptosis, including the activation of the cell cycle, DNA replication, and mismatch repair pathways (Fig. 2c).

A large number of studies have documented the association between TME-infiltrating immune cells and RNA modification. Therefore, we attempted to investigate the functional role of “writers” in TME [31, 32]. To compare the component differences of immune cells among the RNA modification patterns, we used the CIBERSORT method [49], a deconvolution algorithm that uses support vector regression to determine the type of immune cells type in tumors (Table S6-S9). This approach showed that RNA modification “writers” may have a strong correlation with TME cell infiltration (Figure S3B). For example, CSTF1, CSTF3, CPSF3, TRMT6, and TRMT61B were significantly negatively correlated with M2 macrophage differentiation. Differences in TME cell infiltration between the two RNA modification clusters were also analyzed. We observed that the infiltration by M2 macrophages (*p* = 1.2 × 10^− 16^), T regulatory cells (Tregs) (*p* = 2.2 × 10^− 12^), T follicular helper cells (Thf cells) (*p* = 0.0016), and T gamma delta cells (*p* = 0.0062) was higher in Cluster_1. The infiltration of activated DCs (*p* = 9.7× 10^− 5^), natural killer cells (*p* = 0.015), and M1 macrophages (*p* = 0.0068) were higher in Cluster_2 (Fig. 2e). Overall, the RNA modification Cluster_1 was enriched in immunosuppressive cells, e.g., M2 macrophages and Tregs, indicative of poor prognosis (Fig. 2d). Notably, the distribution of the two types of polarization of macrophages differed significantly between the two clusters. The M2 macrophages were significantly enriched in Cluster_1 (Fig. 2e-f), while M1 macrophages were dominant in Cluster_2. In agreement with this conclusion, the analysis of the expression of macrophage markers indicated that M2 macrophage marker genes IL1R1, FIZ1, TGFB1, IL10, and ARG1 were significantly upregulated in Cluster_1 compared to Cluster_2, while M1 macrophage marker genes IL12A, NOS2, IL23A, and IL15RA were significantly downregulated (Fig. 2g). These suggest RNA modification patterns affected the degree of infiltration by specific immune cell types but did not alter the types of infiltrating immune cells.

### Construction of RNA modification “writer” signature

To further characterize the functional role of the two RNA modification patterns identified above, we identified 463 RNA phenotype-related DEGs and performed enrichment analysis. We found that these genes showed enrichment in biological processes, particularly those related to DNA replication, cell cycle, and tRNA metabolic process (Figure S4A). They were also enriched in signaling pathways, particularly the p53 signaling pathway and IL-17 signaling pathway (Figure S4B). To further validate this differential regulation, we performed unsupervised clustering analyses based on the 463 genes related to RNA modification. This analysis classified the patients into two genomic subtypes: gene.cluster_A and gene.cluster_B (Table S3 and S10). Consistent with the clustering of RNA modification patterns (Fig. 2b), 205 of 562 CRC patients were clustered in gene.cluster_A, which is related to Cluster_1, while 357 were clustered in gene.cluster_B, which is related to Cluster_2. In addition, patients in gene.cluster_A had a worse prognosis than patients in gene.cluster_B (Figure S4C; *p* = 0.0021, log-rank test), similarly to patients in Cluster_1 (Fig. 2d).

**Fig. 3:**
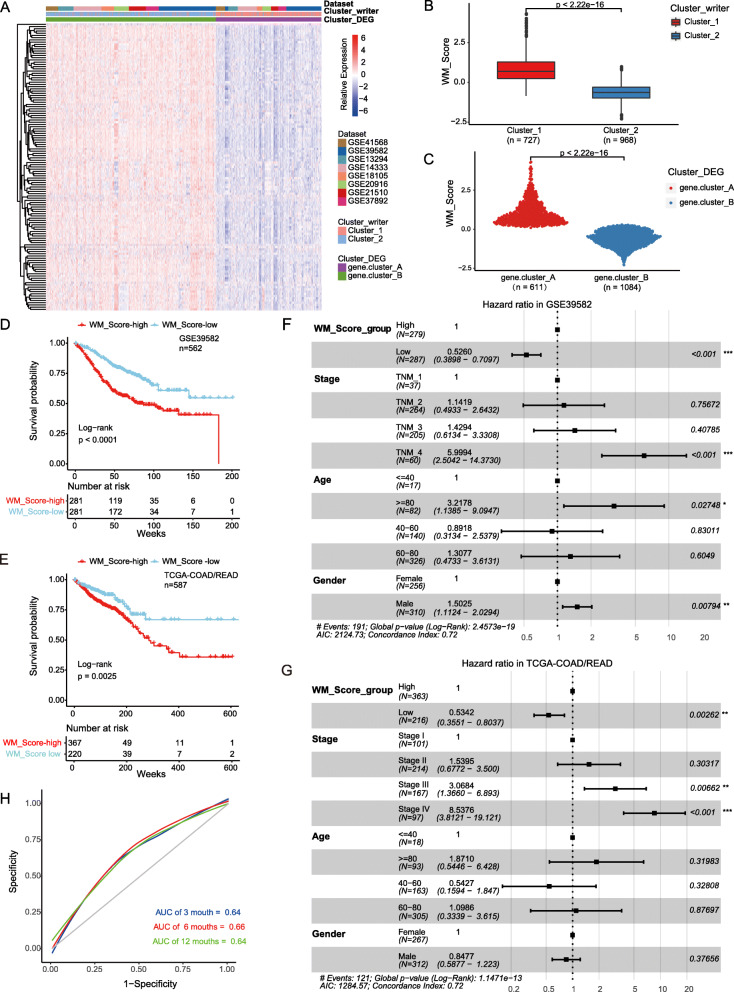
Construction of RNA modification characteristic signature. **a** Unsupervised clustering of the RNA modification phenotype-related genes. The names of CRC cohorts were used as sample annotations. Red, high expression of phenotype-related genes; blue, low expression. **b-c** Differences in the WM_Score between RNA modification patterns (**b**) and gene clusters (**c**) in eight GEO-CRC cohorts. The Wilcoxon test was used to determine the statistical significance of the difference, and *p* < 0.05 was considered statistically significant. **d-e** Kaplan-Meier curves show overall survival in WM_Score-high (red) and -low (blue) in the GSE39582 (**d**) and TCGA-COAD/READ (**e**) cohorts. The grouping of CRC samples is shown at the bottom of the chart. *p* < 0.05 in the two-sided log-rank test was considered statistically significant. **f-g** Multivariate Cox regression model analysis, which included the factors of WM_Score, patient age, gender, TNM status, and patient outcomes in the GSE39582 (**f**) and TCGA-COAD/READ (**g**) cohorts. The length of the horizontal line represents the 95% confidence interval (CI) for each group. The vertical dotted line represents the hazard ratio (HR) of all patients shown by the forest plot. **h** The predictive value of WM_Score in patients among the GSE39582 cohort (AUC:0.64, 066, and 0.64; 3, 6, and 12-months overall survival)

Given the heterogeneity and complexity of RNA modifications, we constructed a DEGs-based score model based on these phenotype-related genes to quantify the RNA modification pattern of individual patients with CRC; this model was termed as the WM_Score (“Writers” of RNA Modification_Score; see Methods). We found that WM_Score of Cluster_1 significantly higher than Cluster_2 (Fig. 3b; Wilcoxon test, *p* < 2.2 × 10^− 16^). In consistence, gene.cluster_A had significantly higher WM_Score than gene.cluster_B (Fig. 3c; Wilcoxon test, p < 2.2 × 10^− 16^). To assess the effect of the WM_Score on TME, we compared the infiltration of immune cells between the WM_Score-low and -high groups. We found that the infiltration of M2 macrophages, Tregs, Tfh cells, and T gamma delta cells was higher in the WM_Score-high group, and the infiltration of activated DCs and M1 macrophages was higher in the WM_Score-low group (Figure S4D).

We performed overlap analysis of these three different classifiers based on the Wayne diagram and the histogram of frequency distribution. As shown in Figure S4, 668 out of 838 (79.72%) samples in the WM_Score high group overlap with the Cluster_1 sample, 798 out of 857 (93.12%) samples in the WM_Score low group overlap with the Cluster_2 sample (Figure S4E-F). Six hundred eleven out of eight hundred thirty-eight (72.91%) samples in the WM_Score high group overlap with samples in the genecluster_A, and 857 (100%) samples in the WM_Score low group overlap with samples in the gene.cluster_B group (Figure S4G-H). The results suggested that these three computational methods of classification have a high degree of coincidence.

To further assess the clinical relevance of the WM_Score, we divided patients into WM_Score-low and -high group with the cutoff value determined by the survminer package. Patients with low WM_Score demonstrated a prominent survival benefit (Fig. 3d; log-rank test, *p* < 1.3 × 10^− 6^). The AUCs of the time-dependent ROC curves for the WM_Score were 0.64, 066, and 0.64 at, respectively, 3, 6, and 12-months overall survival (Fig. 3h). To examine whether the WM_Score could serve as an independent prognostic factor, we performed multivariate Cox regression analysis using the patient clinical characteristics, including age, gender, and TNM status. We found that WM_Score was a robust and independent prognostic biomarker for evaluating patient outcomes (Fig. 3f; HR = 0.5260, 95% CI 0.3898–0.7097, *p* < 0.001). The reliability of the WM_Score was validated using 562 samples of CRC patients from the TCGA (Fig. 3e,g; Table S4). Consistent with these findings, the WM_Score-low group had a better overall survival advantage than the WM_Score-high group in univariate (Fig. 3e; log-rank test, *p* = 0.0025) and multivariate (Fig. 3g; HR = 0.5342, 95% CI 0.3551–0.8037, *p* = 0.00262) Cox regression analysis. These results imply that the WM_Score can reflect the RNA modification patterns and predict the prognosis of CRC patients.

### Molecular subtypes and clinical characteristics associated with WM_Score in CRC

CRC can be divided into four consensus molecular subtypes (CMSs), CMS1–4, with distinct molecular features [50]. They include CMS1 (microsatellite instability) with immune cell infiltration, hypermutated state, unstable microsatellites, and strong immune activation, CMS2 (canonical) with marked activation of WNT and MYC signaling, CMS3 (abnormal metabolism) with evident metabolic dysregulation, and CMS4 (mesenchymal) with prominent TGF-β activation, invasion of stromal cells, and angiogenesis [50]. We have shown that Cluster_1 is accompanied by the activation of EMT, TGF-β, and VEGF signaling pathways (Fig. 2c). Additionally, we calculated the EMT score based on the expression of 25 epithelial marker genes and 52 mesenchymal marker genes [51] and found a positive correlation between the WM_Score and EMT score (Figure S5A; *Rs* = 0.29; *p* = 1.7 × 10^− 8^). Moreover, the EMT score was significantly higher in the WM_Score-high group than in the WM_Score-low group in the TCGA-COAD/READ cohort (Figure S5B; *p* = 5.2 × 10^− 5^).

**Fig. 4:**
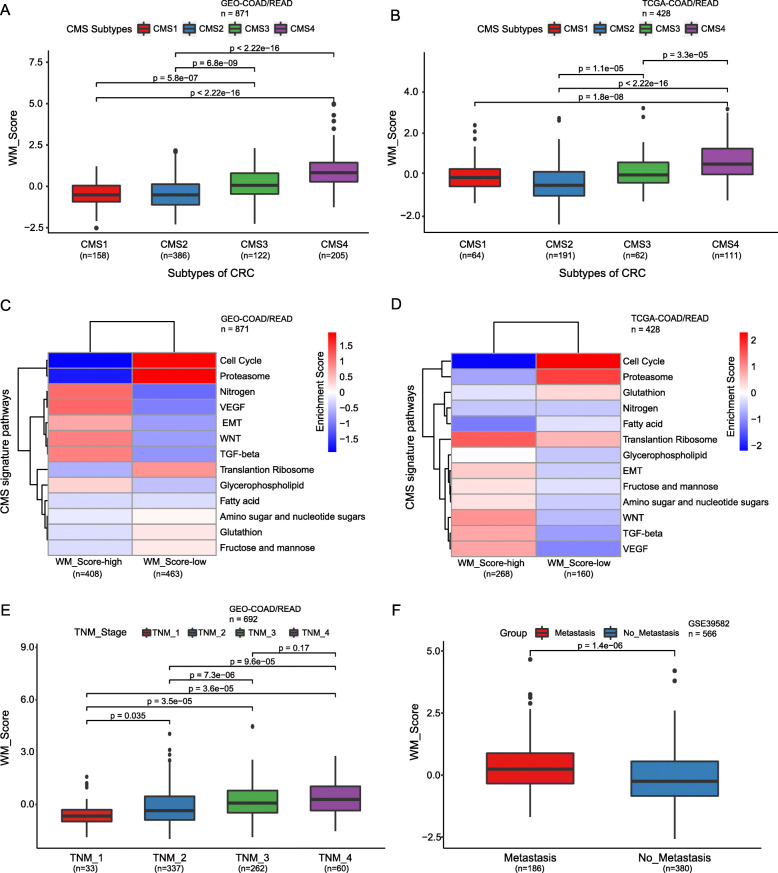
Biological characteristics of CRC associated with the WM_Score. **a-b** WM_Score differences among CMS subtypes of CRC in four GEO-CRC datasets (**a**) and the TCGA-COAD/READ cohort (**b**). **c-d** Heatmap shows the differences in enrichment in the characteristic signaling pathways of CMS subtypes between WM_Score-high and -low groups in four GEO-CRC datasets (**c**) and the TCGA-COAD/READ cohort (**d**). Red, high enrichment score; blue, low enrichment score. **e** WM_Score differences among TNM stages of CRC in eight GEO-CRC datasets. **f** Differences in the WM_Score between metastasis (red) and non-metastasis (blue) group in the GSE39582 cohort. Wilcoxon test was used to assess the difference. The boxes indicate the median ± 1 quartile, with the whiskers extending from the hinge to the smallest or largest value within 1.5× IQR from the box boundaries

To examine the association between the WM_Score and CMS subtypes, we compared the WM_Scores of different CMS subtypes in four GEO datasets (GSE39582, GSE13294, GSE14333, GSE20916) and the TCGA Cohort, respectively (Table S3–4). We found that WM_Scores varied significantly among CMS subtypes, with the CMS4 group showing the highest score (Fig. 4a-b). Also, the distribution of CMS subtypes was significantly different between the WM_Score-high and -low groups. The CMS4 subtype was more frequent in the WM_Score-high group, while the CMS1/2 subtype was predominant in the WM_Score-low group (Figure S5C-D). We further analyzed the signaling pathways characteristic of the different CMS subtypes. The signaling pathways activated mostly in the CMS4 samples were WNT, TGF-β, VEGF, and EMT signaling pathways, while the characteristic signaling pathways activated in CMS1/2 were those related to the cell cycle and proteasome (Figure S5E-F). Consistently, in both cohorts, the enrichment score of CMS4-related signaling pathways was significantly higher in the WM_Score-high group, while the enrichment score of CMS1/2-related signaling pathways was significantly higher in the WM_Score-low group (Fig. 4c-d).

CMS subtypes are reflected in tumor progression and clinical outcome. CMS4 tumors tend to be diagnosed at more advanced stages (Figure S5G) and are associated with worse overall survival (Figure S5H; log-rank test, *p* = 0.01). We further documented that the WM_Score is different among tumor stages and is higher in more advanced CRC (Fig. 4e), suggesting the involvement of parameters comprising the WM_Score in tumor progression. Although improved treatment strategies involving surgery and chemo- and radiotherapy have increased the overall survival rates in patients with early stages of CRC, 40–50% of CRC patients present with metastasis either at the time of diagnosis or as a recurrent disease after a curative therapy [52]. Most CRC patients with distant metastasis are not suitable candidates for conventional treatment and exhibit a poor 5-year survival rate of < 10% [53]. The correlation between the WM_Score and CMS4 subtype and between the WM_Score and CRC stage suggests that the WM_Score might be associated with patient survival by reflecting tumor metastasis. WM_Score was significantly higher in metastatic than in non-metastatic CRC patients (Fig. 4f). These results suggest that the WM_Score correlates closely with CMS subtypes, and a high WM score may indicate a poor prognosis by being associated with the activation of EMT, TGF-β, and other signaling pathways mediating tumor metastasis.

### WM_Score involved in transcriptional and post-transcriptional regulation

**Fig. 5:**
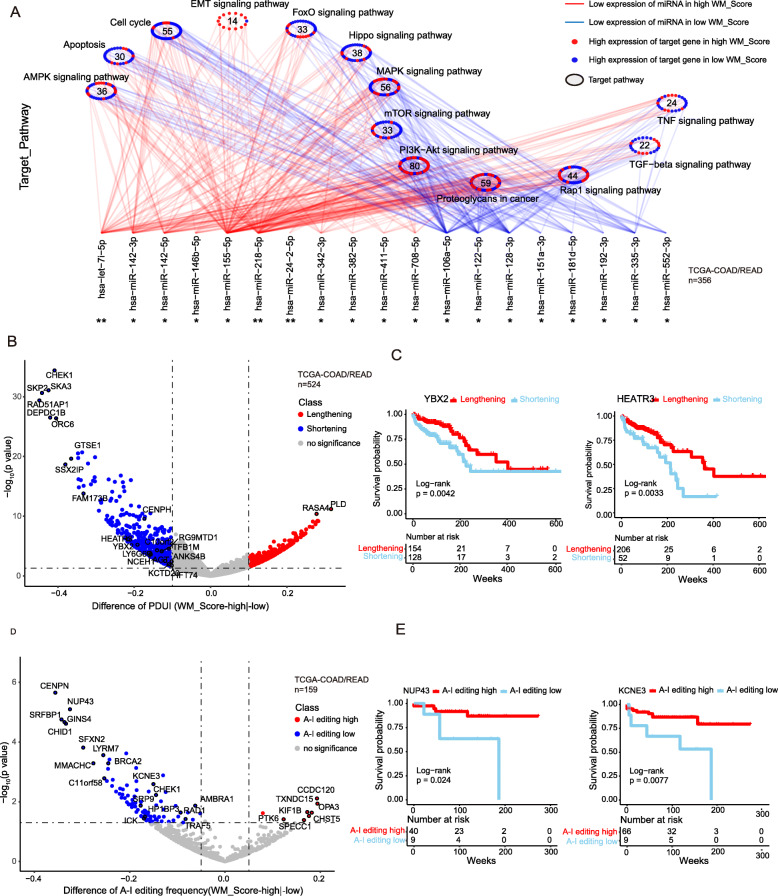
Transcriptional and post-transcriptional characteristics associated with the WM_Score. **a** Differences in miRNA-targeted signaling pathways in the TCGA-COAD/READ cohort between the WM_Score-high and -low groups. The red line represents a low expression of miRNA in the high WM_Score group, and the blue line represents a high expression of miRNA in the low WM_Score group. Red dots correspond to miRNA-targeted genes highly expressed in the high WM_Score group, and blue dots correspond to miRNA-targeted genes highly expressed in the low WM_Score group. The circle represents a signaling pathway enriched with targeted genes. **b** The differences in the distal poly(A) site usage index (PDUI) of each gene between WM_Score-high and -low groups. Red, PDUI lengthening; blue, PDUI shortening; Grey, no significant change in PDUI. **c** Kaplan-Meier curves show overall survival between PDUI lengthening (red) and PDUI shortening (blue) of YBX2 and HEATR3. The grouping of CRC samples is shown at the bottom of the chart. *p* < 0.05 in the two-sided log-rank test was considered statistically significant. **d** The frequency of A-I editing was compared between WM_Score-high and -low groups. Red, high A-I editing; blue, low A-I editing; grey, no significant change in A-I editing. **e** Kaplan-Meier curves show overall survival between PDUI lengthening (red) and PDUI shortening (blue) of NUP43 and KCNE3. The grouping of CRC samples is shown at the bottom of the chart. *p* < 0.05 in the two-sided log-rank test was considered statistically significant

WM_Score is an assessment model based on the expression of 26 RNA modification “writers”, which regulate post-transcriptional modifications, including RNA transport, localization, translation, and other biological processes. To further assess the power of WM_Score in the interpretation of transcriptional and post-transcriptional events, we focused on RNA modification “writers”-related processes (e.g., APA, RNA editing). Given that transcripts processed by APA have a short 3’UTR, thus tolerating the regulation of miRNAs [54], we hypothesized that RNA modification patterns were associated with distinct miRNA characteristics under the action of APA modification “writers”. We first analyzed differences in miRNAs expression among different RNA modification patterns in the TCGA-COAD/READ cohort. Thirty-nine miRNAs significantly differentially expressed between WM_Score-high and -low groups were screened out, and enrichment analysis of the signaling pathways of their target genes was performed (Table S11). The miRNA-targeted genes were significantly correlated with cell cycle, apoptosis, EMT, PI3K-Akt, and other signaling pathways, and the targeted genes were differentially expressed between the two groups. Thirteen out of 14 miRNA-targeted-DEGs in the EMT signaling pathway were highly expressed (Fig. 5a). In contrast, target genes of a group of miRNAs with lower expression in the WM_Score-low group were upregulated and were significantly enriched in the cell cycle and apoptosis signaling pathway. Specifically, 48 out of 55 miRNA-targeted DEGs in the cell cycle, 24 out of 30 miRNA-targeted DEGs in apoptosis, 25 out of 33 miRNA-targeted DEGs in the mTOR signaling pathway showed enrichment in the WM_Score-low group (Fig. 5a). These analyses suggested that the WM_Score is associated with miRNA expression and the regulation of signaling pathways.

To explore the functional role of RNA modification writers, we analyzed the APA and A-I editing events of each gene in the TCGA-COAD/READ cohort to observe the post-transcription characteristics. We identified the genes with the differences in APA between different RNA modification patterns and compared the survival associated with these genes to determine whether the length of 3’UTR affects the survival of CRC patients (Figure S6A; Table S12–13). Most of the genes with shortening APA events were enriched in the WM_Score-low group and were associated with shorter survival (Fig. 5b and S6A). Akt was associated with the telomerase-reverse transcriptase catalytic subunit HEATR3, which may be activated by Akt in melanoma [55, 56]. A rescue study indicated that LINC00958 regulates the proliferation, motility, and EMT of oral squamous cell carcinoma cells through YBX2 [57]. HEATR3 (Diff = − 0.22; *p* = 7.61 × 10^− 7^) and YBX2 (Diff = − 0.19; *p* = 5.67 × 10^− 6^) transcripts exhibited statistically significant shortening, which was associated with worse survival of CRC patients (Fig. 5c; HEATR3: log-rank test, *p* = 0.0033; YBX2: log-rank test, *p* = 3.0 × 10^− 4^). We raised the possibility that in the WM_Score-high group, due to the shortening of HEATR3 and YBX2, which, in turn, shorten the 3’UTR, miRNA may not be able to target the corresponding gene, resulting in the activation of gene expression and contributing to the initiation and development of CRC.

We identified the genes with differences in A-to-I editing between WM_Score-high and -low groups in 159 TCGA-READ/COAD samples (Table S14–15). The A-to-I editing differential genes identified were less due to relatively low read coverage of RNA-seq data in CRC, with fewer informative editing sites [58]. An increasing amount of evidence shows that ADAR1-mediated A-to-I editing can modify the 3’UTR region of mRNA, affecting the binding of miRNAs [59]. The genes with lower A-to-I editing rate were enriched in the WM_Score-low group and were associated with worse survival of CRC patients (Fig. 5d and S6B). NUP43 is a stable component of the Nup107–160 complex, which may regulate the malignant transformation of aged cells by enabling the translocation of the phosphorylated extracellular signal-regulated kinase (ERK) to the nucleus [60]. Reduction in NUP107 attenuates growth factor signaling in senescent eukaryotic cells, triggering their apoptosis [61]. The potassium channel KCNE3 is a VEGFA-inducible gene selectively expressed in vascular endothelial cells [62]. KCNE3 is suppressed in tumors responding to the VEGFA blockade [63]. The A-to-I editing of NUP43 (Diff = − 0.33; *p* = 8.0 × 10^− 6^) and KCNE3 (Diff = − 0.15; *p* = 0.0026) is associated with shorter survival time of CRC patients (Fig. 5e; NUP43: log-rank test, *p* = 0.024; KCNE3: log-rank test, *p* = 0.0077). The difference in the rate of A-to-I editing of these genes between WM_Score-high and -low groups may be regulated by miRNA via the editing of the 3’UTR regions, thus affecting the occurrence and development of CRC.

### Potential therapeutic value of the WM_Score

**Fig. 6:**
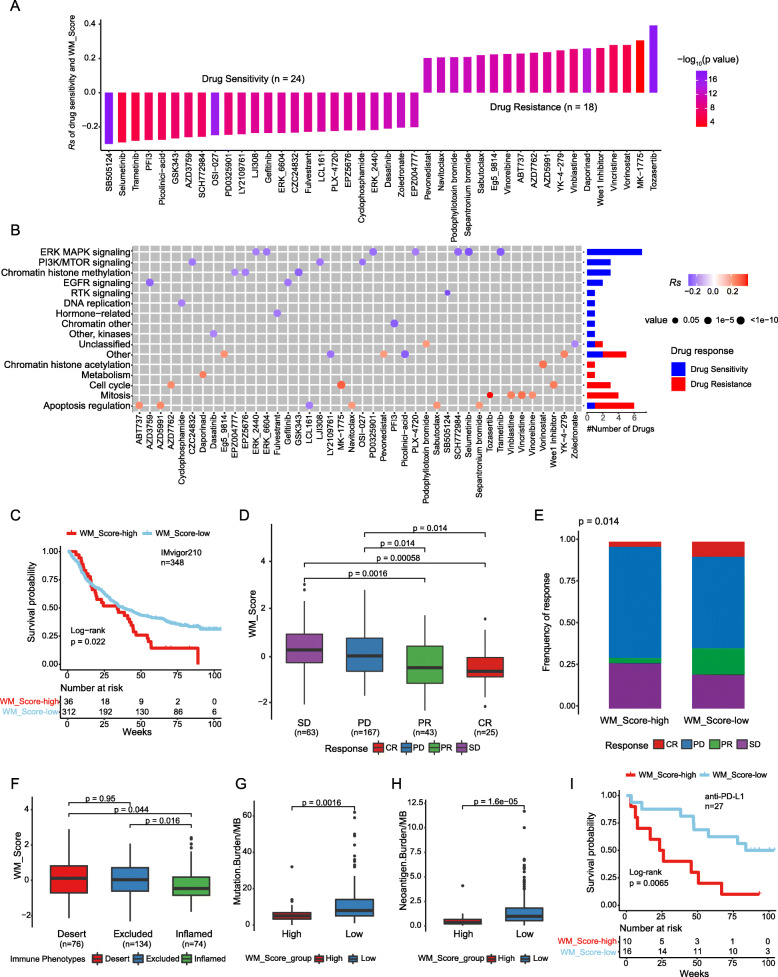
The relationship between WM_Score and drug sensitivity and efficacy of immunotherapy. **a **The correlation between WM_Score and drug sensitivity evaluated by the Spearman analysis. Each column represents a drug. The brightness of the column indicates the significance of the correlation. The height of the column indicates the correlation, indicates that WM_Score related to drug resistance (*Rs* > 0) or drug sensitive (*Rs* < 0) to WM_Score. **b** Signaling pathways targeted by drugs that are resistant (red) or sensitivity (blue) to the WM_Score. Drug names are listed on the horizontal axis and the signaling pathway targeted by the drug on the vertical axis. The bar graph on the right shows the number of drugs targeting each signaling pathway. The size of the point indicates the significance of the correlation. **c, i** Kaplan-Meier curves show overall survival in the WM_Score-high (red) and -low (blue) subgroups after the PD-L1 blockade immunotherapy in the IMvigor210 (**c**) and anti-PD-L1 (**i**) cohort. The grouping of patients is shown at the bottom of the chart. *p* < 0.05 in the two-sided log-rank test was considered statistically significant. **d** The difference in the WM_Score between distinct clinical outcomes of anti-PD-L1 treatment in the IMvigor210 cohort. **e** The proportion of patients in the IMvigor210 cohort with different responses to PD-L1 blockade immunotherapy. The fisher.test was used to determine the statistical significance of the difference. SD, stable disease; PD, progressive disease; CR, complete response; PR, partial response. **f** The difference in the WM_Score among immune phenotypes, including the inflamed (green), excluded (blue), and desert (red) immune type in the IMvigor210 cohort. (G-H) Differences in TMB (**g**) and neoantigen burden (**h**) between WM_Score-high (red) and -low (blue) groups in the IMvigor210 cohort. Wilcoxon test was used to assess the difference. The boxes indicate the median ± 1 quartile, with the whiskers extending from the hinge to the smallest or largest value within 1.5× IQR from the box boundaries

To further understand the effects of the WM_Score on drug response, we assessed the association between the WM_Score and the response to drugs in cancer cell lines. Using the Spearman correlation analysis, we identified 42 significantly correlated pairs between WM_Score and drug sensitivity in the Genomics of Drug Sensitivity in Cancer (GDSC) database [64] (Fig. 6a,Table S16). Among them, 24 pairs showed that drug sensitivity correlated with the WM_Score, including the MEK inhibitor Selumetinib (*Rs* = − 0.29, *p* = 1.31 × 10^− 15^), mTOR inhibitor LJI308 (*Rs* = − 0.24, *p* = 8.21 × 10^− 10^), and EGFR inhibitor AZD3759 (*Rs* = − 0.26, *p* = 4.12 × 10^− 13^). Eighteen pairs exhibited drug resistance correlated with the WM_Score, including cell cycle checkpoint kinase inhibitor AZZD7662 (*Rs* = 0.23, *p* = 9.9 × 10^− 11^) and Bcl-2 inhibitor AZD5991 (*Rs* = 0.24, *p* = 1.19 × 10^− 9^). Further, we analyzed the signaling pathways of the genes targeted by these drugs. We found that drugs whose sensitivity was associated with WM_Score-high were mostly targeting MAPK, mTOR, and VEGF signaling pathways. In contrast, the drug whose sensitivity was associated with WM_Score-low were targeting apoptosis and cell cycle signaling pathway (Fig. 6b). Together, these results imply that RNA modification patterns are correlated with drug sensitivity. Thus, the WM_Score may be a potential biomarker for establishing appropriate treatment strategies.

### The WM_Score model predicts response to immunotherapy with PD-L1 blocker

A major effort has been made to identify biomarkers to predict the response to immunotherapy, including tumor mutation burden (TMB) and the expression of PD-L1 protein [65–67]. Considering that WM_Score appears to be associated with the immune microenvironment of the tumor (Figure S4D), we examined the power of the WM_Score to predict the response of patients to ICB therapy. This analysis was based on two immunotherapy cohorts (Fig. 6c-i). We found that patients with WM_Score-low exhibited significant clinical benefits and had a markedly prolonged overall survival in both anti-PD-L1 cohorts, including the IMvigor210 cohort [68] (Fig. 6c; log-rank test, *p* = 0.022) and the bladder cancer cohort [69] (Fig. 6i; log-rank test, *p* = 0.0065). The 348 patients of the IMvigor210 cohort exhibited different degrees of response to anti-PD-L1 blocker, including complete response (CR), partial response (PR), stable disease (SD), and progressive disease (PD). The CR patients showed the lowest WM_Score than patients with other types of responses (Fig. 6d). The chi-squared test performed between WM_Score-low and -high groups also showed significantly better therapeutic outcomes in WM_Score-low patients (Fig. 6e; *p* = 0.014). We analyzed the WM_Score of the three immune subtypes of IMvigor210, including “immune inflamed”, “immune excluded”, and “immune desert” [70] (Fig. 6f), and observed that the WM_Score of the “immune inflamed” type was lower than in the other two groups. In addition, the TMB and neoantigen burden were significantly higher in the WM_Score-low group than in the WM_Score-high group (Fig. 6g-h), which may explain, at least in part, the survival advantage and the greater benefit of the ICB treatment in the WM_Score-low group.

We have also found that the activation of M2 macrophages and TME of stromal cells was significantly higher in WM_Score-high tumors, and these processes may mediate the immune tolerance of tumors. This result suggested that WM_Score-high tumors may represent “cold tumors” characterized by resistance to immunotherapy. In summary, the performed analyses suggest that the established “writers” of RNA modification score model might improve the selection of drugs for CRC and the prediction of response to anti-PD-L1 immunotherapy.

## Discussion

Increasing evidence shows that RNA modifications play an indispensable role in inflammation, innate immunity, and antitumor activity through interaction with various “writers”. While most studies have focused on a single type of RNA modification “writer’, the mutual relationships and functions of multiple types of “writers“ in cancer are not fully understood. Here, We revealed global alterations of m^6^A, m^1^A, APA, and A-to-I RNA editing enzymes (Fig. S7A) at transcriptional and genetic levels and their mutual correlation in CRC (Fig. S7B). Then we identified two distinct RNA modification patterns based on 26 RNA modification enzymes, defined two RNA-modification-related subtypes of CRC, and constructed a scoring model, WM_Score, to assess the efficacy of RNA modification “writers” in individual patients (Fig. S7C). The WM_Score-high subtype is associated with worse prognosis (Fig. S7D). The abundance of immune cells in the tumor microenvironment was significantly different between the two CRC subtypes, and WM_Score-high subtype is associated with higher infiltration of inhibitory immune cells, including M2 macrophages, plasma cells, Tregs, and Tfh cells (Fig. S7E). This CRC subtype is also characterized by a significant activation of EMT, TGF-β, WNT, and VEGF signaling pathways (Fig. S7F), which are conducive to tumor invasion into the stroma and formation of blood vessels [71].

EMT is involved in cancer cell metastasis and drug resistance [72], and M2 macrophages suppress T cell proliferation and differentiation, promoting the proliferation of tumor cells and tumor stromal angiogenesis [73]. A previous study showed that the M2 polarization of tumor-associated macrophages (TAMs) is associated with EMT progression and increased migration and invasion of tumor cells [74, 75] in later stages of cancer. TGF-β signaling may enhance tumor progression by promoting cell proliferation and EMT and suppressing immune function [76, 77]. Over-activation of the WNT/β-catenin pathway promotes EMT-associated dedifferentiation taking place at the invasive front of colorectal tumors [78]. The activation of TGF-β and WNT signaling pathways in the WM_Score-high subtype is likely to promote the polarization of TAMs to the M2 phenotype in the tumor microenvironment, thus promoting the activation of EMT and VEGF signaling pathway. These changes may increase angiogenesis in the tumor microenvironment, potentiating the invasion and metastasis of colorectal cancer cells. On the contrary, WM_Score-low subtype patients had significantly longer survival and a higher infiltration of memory CD4^+^ T cells, M1 macrophages, and DCs, also upregulated signaling pathways of apoptosis, DNA damage repair, and cell cycle. RNA modification writers, e.g., NUDT21, can switch APA sites in genes regulating the cell cycle, apoptosis, and metabolism, resulting in the inhibition of tumor cell proliferation, metastasis, and tumorigenesis [32, 79]. M1 macrophages secrete IL-12, IL-16, INF-γ, and other proinflammatory cytokines, activating the inflammatory response and eliminating tumor cells [73]. These properties are enriched in the WM_Score-low group, suggesting that RNA modification writers may regulate post-transcriptional events (Fig. S7G) involved in immune infiltration, cell cycle, apoptosis, and other signaling pathways, thus modulating tumorigenesis.

Additionally, RNA modifications affect regulatory genes regulating EMT, cell cycle, and apoptosis by mediating the differential expression of miRNA, e.g., let-7i-5p and let-142-3p. Our study identified differences in miRNA expression mediated by RNA modification patterns, target genes, and signaling pathways (Fig. S7G). In the high WM_Score subtype, the EMT and PI3K-Akt signaling pathways targeted by the differentially expressed miRNA were significantly activated. In contrast, in the WM_Score-low subtype, the signaling pathways targeted by differentially expressed miRNAs were mostly related to the cell cycle and apoptosis.

The link between EMT and the drug resistance of cancer cells has been postulated in the early 1990s [80]. Since then, it has been increasingly recognized that cancer drug resistance is frequently accompanied by EMT in diverse types of cancer, including pancreatic, bladder, and breast cancer [81]. For instance, TGF-β, a well-studied EMT-related cytokine, was reported to be related with drug resistance in the 1990s. Teicher and coworkers demonstrated that TGF-β-neutralizing antibodies restore drug sensitivity in the tumors resistant to alkylating agents [82]. Subsequent studies documented that TGF-β induces EMT, leading, in turn, to drug resistance. TGF-β signaling can induce EMT through GTPases, and PI3K, MAPK/ERK, WNT, and AKT/mTOR pathways [83, 84], which ultimately activate EMT transcription factors (EMT-TFs).

Finally, we showed the potential therapeutic effects of RNA modification writers in CRC (Fig. S7H). WM_Score was associated with resistance to drugs targeting the cell cycle and apoptosis pathways, and with sensitivity to drugs targeting ERK/MAPK, PI3K/mTOR, and EGFR signaling pathways. These results imply that patients with higher WM_Score may benefit from drugs targeting these signaling pathways, rather than from drugs targeting the cell cycle or apoptosis pathways. RNA modification patterns might be regarded as an adequate “predictor” to evaluate the clinical outcome of chemotherapy or targeted therapies. The WM_Score could also predict the response of patients to anti-PD-L1 immunotherapy (Fig. S7H). By identifying different immune phenotypes of tumors and enabling personalized cancer immunotherapy, our findings provide new possibilities for improving the outcome of immunotherapy for CRC.

## Conclusions

Our systematic, integrated analysis of four types of RNA modification “writers” revealed an extensive regulatory mechanism by which they affect tumor microenvironment and their relationship with CRC prognosis. We constructed WM_Score model documented the cross-talk and regulatory roles of the “writers” in transcription and post-transcriptional events and identified their therapeutic utility in targeted therapy and immunotherapy. This work highlights the crucial clinical implications of the cross-talk of RNA modifications and helps develop personalized immune therapeutic strategies for CRC patients.

## Methods

### Data collection and processing

The workflow of our study was shown in Figure S1A. Public gene expression data and complete clinical annotations from the same sequencing platform were retrieved in Gene-Expression Omnibus (GEO) and the Cancer Genome Atlas (TCGA) database. mRNA expression, miRNA expression, somatic mutation, SCNAs, and clinical data, including tumor stage, histology subtype, gender and overall survival times were obtained from TCGA database ﻿(https://portal.gdc.cancer.gov/). Eight GEO colorectal cancer cohorts (GSE41568, GSE39582, GSE13294, GSE14333, GSE18105, GSE20916, GSE21510, GSE37892) and TCGA-COAD/READ cohort were included for further analysis. The data information is summarized in Table S2. The “ComBat” algorithm of sva Package [85] was used to correct the batch effect caused by non-biotechnological bias. The data was analyzed using R (version 3.6.2) and R Bioconductor packages.

We collected datasets with immunotherapy. The IMvigor210 cohort and bladder cancer with anti-PD-L1 cohort were included in the study of the relationship between WM_Score and immunotherapy prognosis. The IMvigor210 cohort [68]: Intervention treatment of advanced urinary tract transitional cell carcinoma (atezolizumab, anti-PD-L1 antibody). IMvigor210 cohort with the expression of the data and detailed clinical notes are downloaded from http://research-pub.gene.com/IMvigor210CoreBiologies. Expression and clinical information of bladder cancer with anti-PD-L1 cohort downloaded from 10.5281/zenodo. 546,110 [69].

### Clustering expression pattern of 26 RNA modification “writers”

Unsupervised clustering algorithm was applied to cluster analysis of RNA-modified “writers” in 1695 colorectal cancer samples. RNA modification “writer” consists of 7 m^6^A modification enzymes (METTL3, METTL14, WTAP, RBM15, RBM15B, ZC3H13, and KIAA1429), 4 m^1^A modification enzymes (TRMT61A, TRMT61B, TRMT10C, and TRMT6),12 APA modification enzymes (CPSF1-4, CSTF1/2/3, PCF11, CFI, CLP1, NUDT21, and PABPN1) and 3 A-I modification enzymes (ADAR, ADARB1, and ADARB2). Unsupervised clustering was applied to detect the robust clustering of colorectal cancer. We used the Consensus-Clusterplus package for the above steps, and conduct 1000 repetitions to ensure the stability of the classification [86, 87].

### Gene set variation analysis (GSVA) and functional annotation

In order to study the differences of RNA modification patterns in biological processes, we used “GSVA” R package to conduct GSVA enrichment analysis [42]. The gene set “c2.cp.kegg.v7.1” and “ h.all.v7.2 “ for GSVA analysis was downloaded from the MSigDB database. The clusterProfiler R Package was used to functionally annotate 26 RNA modification enzyme genes [88].

### Calculation of TME cell invasion abundance

We use CIBERSORT algorithm (https://cibersort.stanford.edu/) to quantify the relative abundance of 22 types of immune cells in colorectal cancer with parameters as follows: the input mixture matrix is our gene expression matrix, the input of gene signature reference for 22 immune cell types from Newman et al. [49], 100 times for permutation test, and RNA-seq data without quantile normalization, while microarray data with quantile normalization.

### Constructing the WM_Score scoring system to evaluate individual CRC

1. Identify RNA phenotype-related DEGs. The Limma R package estimates mean-variance relationships prior to linear modeling by converting read count data to log2 transformation Fragments Per Kilobase Million (FPKM), determines the weight of each observed value through the voom function, then applies the data to linear modeling, and uses empirical Bayesian statistics to analyze the DEGs between the RNA modification patterns. We used a univariate Cox regression model to calculate the risk ratio (HR) of DEGs. Then DEGs related to survival were extracted to construct a scoring system.
2. Enrichment analysis of DEGs. Enrichment analysis and functional annotation of DEGs were performed using the clusterProfiler R package [88]. “org.hs.eg.db” was used as annotation to carry out enrichment analysis of GO and KEGG in gene set. DEGs was used as input gene set, *p* value was calculated by ORA (Over-Representation Analysis) [89], and used Benjamini and Hochberg adjustment for false discovery rate (FDR) and considered an FDR < 0.05.
3. Construction of scoring system. After obtaining the prognostic value of each gene signature score, we applied a method similar to GGI [90] to define the WM_Score of each patient: *WM*_*Score*_ = (*beta*_*i*_ × *Exp*_*i*_ ),where i means the RNA modification phenotype-related genes.

### Calculating the EMT score

We obtained epithelial-to-mesenchymal transition gene signatures from Mak et al [51], including 25 epithelial and 52 mesenchymal marker genes. The EMT score for each sample was estimated as $\sum \limits_i^N\frac{M^i}{N}-\sum \limits_j^n\frac{E^j}{n}$, as described in a previous study [51], where *M* and *E* represent the expression of the mesenchymal gene and epithelial gene, respectively, and *N* and *n* respectively represent the number of mesenchymal genes and epithelial genes.

### Comparison of transcription and post-transcriptional events between WM_Score-high and -low groups

#### The association of WM_Score and miRNA

The expression of miRNA in CRC were obtained from TCGA. Analyzing the differentially expressed miRNAs between the WM_Score-high and low groups, the targeted signaling pathways of differentially expressed miRNAs were enriched by KEGG enrichment analysis. To compare the expression of miRNA between WM_Score-high and low-groups, we used the Wilcoxon test and used Benjamini and Hochberg adjustment for FDR and considered an FDR < 0.05 as statistical significance.

#### The association of WM_Score and APA events

APA in CRC were obtained from The Cancer 3′ UTR Atlas (TC3A, http://tc3a.org) [91, 92], which utilized the well-established algorithm DaPars (https://github.com/ZhengXia/DaPars) to identify the alternative proximal polyA site and calculate the Percentage of Distal polyA site Usage Index (PDUI) for each transcript. The alteration of APA usage in each tumor can be quantified as a change in PDUI, which is capable of identifying 3’UTR lengthening (positive index) or shortening (negative index). To compare PDUI between WM_Score-high and -low groups, we used the t test and used Benjamini and Hochberg adjustment for FDR and considered an FDR < 0.05 and PDUI difference > 0.1 as statistical significance.

#### The association of WM_Score and A-to-I editing

A-to-I RNA editing profile in CRC were obtained from Han et al. [58]. Those editing sites with at least three edited reads in at least 3 samples per tissue type were considered to be detected RNA editing sites. To ensure adequate statistical power, they were further identified as the informative RNA editing sites among the detected RNA editing sites by requiring at least 30 samples (including normal samples if available) with coverage ≥10 in a tissue/tumor type. We used the t test to detect RNA editing sites with differential editing between WM_Score-high and -low groups and defined significantly differential editing sites as p value < 0.05 and difference > 5%.

#### The clinical outcome of WM_Score associated APA events and A-to-I editing

To characterize the clinical relevance of APA and RNA editing sites affected by WM_score, we performed the univariate Cox regression analysis to identify the RNA editing level or PDUI that was significantly correlated with patient survival, and considered *p* < 0.05 as statistical significance. We also divided patients into two groups based on the PDUI of APA events or the A-to-I editing rate, and used the Kaplan-Meier curve and the log-rank test to determine the significance of the differences.

### Association analysis of WM_Score and drug sensitivity

The transcription profiles for about 1000 cancer cell lines, drug response measurements as AUC for antitumor drugs in cancer cell lines, and targets/pathways of drugs are downloaded from Genomics of Drug Sensitivity in Cancer (GDSC, http://www.cancerrxgene.org/downloads) [64]. We performed Spearman correlation analysis to calculate the correlation between drug sensitivity and WM_Score, and considered |*Rs*| > 0.2 and used Benjamini and Hochberg adjustment for FDR and considered an FDR < 0.05 as significant correlation.

### Statistical analysis

Spearman and distance correlation were used to calculate the correlation coefficient of RNA modification “writers” expression. Wilcoxon test was used to compare the differences. Receiver operating characteristic (ROC) curve was used to verify the validity of the model.

Based on the correlation between WM_Score and patient survival, survminer package was used to determine the cutoff point of survival information for each dataset. The “surv-cutpoint” function was used to dichotomy WM_Score, and all potential cutting points were repeatedly tested to find the maximum rank statistic, and then the patients were divided into the WM_Score-high group and the WM_Score-low group according to the maximum selected log-rank statistics, so as to reduce the calculated batch effect. Survival curves for prognostic analysis were generated using the Kaplan-Meier method, and the log-rank test was used to determine the significance of the differences. Univariate Cox regression model was used to calculate the hazard ratio (HR) between differentially expressed genes and “writers”. To assess whether WM_Score is an independent predictor, we consider age, gender, and stage as variables to perform multivariate Cox regression model analysis. All statistical analysis was two-side and considered *p* < 0.05 as statistical significance.

## Supplementary Information

**Additional file 1 : Figure S1**. Overview of study design. (A) Flowchart of the steps in the performed analyses. **Figure S2.** Analysis of mutation frequency and CNV in TCGA- COAD/READ. (A) The mutation frequency of RNA modification “writers” among 33 cancer types in the TCGA cohort. The horizontal axis represents cancer types, and the number of samples is given in the parentheses. The vertical axis lists the names of the genes. (B) Comparison of GSEA enrichment analysis between “writers” mutation samples and non-mutation samples. NES, Normalized enrichment score. (C) The distribution of correlation coefficient between “writers” expression and CNV in CRC. |*Rs*| > 0.3 and *p* value < 0.05 indicates that “writers” expression is related to CNV. (C) The expression of “writers” among CNV groups in CRC. The sample size for each group based on the CNV alteration (CFI, CNV_loss/ CNV_gain/ normal/ none_CNV = 138/18/54/305; METTL14, 143/19/54/299; RBM15: 125/17/54/319; ADARB1: 138/26/54/297; TRMT61A: 151/33/54/277; CPSF2: 155/33/54/273; PABPN1: 146/38/54/277; METTL3: 146/38/54/277). Wilcoxon test was used to assess the difference. The boxes indicate the median ± 1 quartile, with the whiskers extending from the hinge to the smallest or largest value within 1.5× IQR from the box boundaries. **Figure S3**. Biological characteristics of RNA modification “writers”. (A) Association of gene expression for 26 RNA modification “writers” with patient overall survival times based on Univariate Cox regression analysis in GSE39582 cohort. (B) Heatmap shows the positive (red) and the negative (blue) correlation between TME infiltration and WM_Score in CRC. *p < 0.05, **p < 0.01, and ***p < 0.001, as determined by the Spearman correlation analysis. **Figure S4.** Enrichment analysis of differentially expressed genes and the relationship between survival and the WM_Score. (A-B) GO (A) and KEGG (B) enrichment analysis of the 463 DEGs. The x-axis indicates gene counts within each GO term. The brightness of the column color represents the statistical significance of enrichment. (C) Kaplan-Meier curves comparing overall survival between two DEG clusters, gene.cluster_A (red) and gene.cluster_B (blue), in the GSE39582 cohort. The grouping of CRC samples is shown under the Kaplan-Meier plot. *p* < 0.05 in the two-sided log-rank test was considered statistically significant. (D) Heatmap shows the differences in TME infiltration between WM_Score-high and -low groups in the GEO-CRC cohort. Red, high enrichment score; blue, low enrichment score. (E-F). Overlap (E) and frequency (F) of classifiers of WM_Score-high/−low and Cluster_1/2 in CRC. (G-H). Overlap (G) and frequency (H) of classifiers of WM_Score-high/−low and gene.cluster_A/B in CRC. The Fisher test was used to determine the statistical significance of the difference. **Figure S5.** Relationship between the WM_Score and the molecular subtype of CRC. (A) Correlation between the EMT score and WM_Score in the TCGA-COAD/READ cohort by Spearman analysis. (B) The difference of EMT scores in WM_Score-high (red) and -low (blue) groups in the TCGA-COAD/READ cohort. (C-D) Distribution of CMS subtypes within WM_Score-high and -low groups in four GEO-CRC datasets (C) and the TCGA-COAD/READ cohort (D). (E-F) Enrichment in signaling pathways in CMS subtypes in four GEO-CRC datasets (E) and the TCGA-COAD/READ cohort (F). (G) Distribution of TNM stage within CMS subtypes in four GEO-CRC datasets. Statistical significance (*p* < 0.05) was calculated using the fisher.test. (H) Kaplan-Meier curves show the difference in overall survival between two RNA modification patterns, WM_Score-high (red) and -low (blue), in the GSE39582 cohort. The grouping of CRC samples is shown below. **Figure S6.** The length of APA PDUI gene affects the survival prognosis of CRC. (A-B) The bar graphs show the difference between WM_Score-high and -low groups in PDUI (A) and A-I editing (B). The forest plots show univariate Cox regression analyses for PDUI differential genes (A) and A-I editing differential genes (B) between WM_Score-high and -low group.  **Figure S7.** Graphic summary. (A) m^6^A, m^1^A, APA, and A-to-I RNA editing enzymes. (B) Genetic alterations (left panel), transcriptional alterations (intermediate panel), and mutual correlation (right panel) of four types of RNA modification "writers" in CRC. (C) Two distinct RNA modification patterns based on 26 RNA modification enzymes, and constructed a scoring model WM_Score. (D) Clinical relevance of WM Score. (E) Distinct patterns of WM_Score associated with immune infiltration. (F) Molecular subtypes associated with WM_Score in CRC. (G) WM_Score involved in transcriptional and post-transcriptional regulation. (H) Therapeutic ability of the WM_Score.

**Additional file 2 Supplementary Table 1.** Summary of RNA Modification Writers. The annotation of 26 RNA modification “writers” based on the published data. **Supplementary Table 2.** Clinical information of CRC cohorts from GEO/TCGA. The accession number, platform of microarray, the number of tumor and normal samples, clinical characteristics (stage, gender), citation of CRC cohort. **Supplementary Table 3.** Samples clustering in eight GEO-CRC cohorts. The detailed information of eight GEO-CRC Cohorts with different group methods, including clustering, CMS subtypes, and WM_Score. **Supplementary Table 4.** Group information of samples in TCGA-COAD/READ cohorts. The WM_Score and the classification of WM_Score types and CMS subtypes in TCGA-COAD/READ cohorts. **Supplementary Table 5.** Enrichment score of KEGG pathways in eight GEO-CRC cohorts. The table shows enrichment score of KEGG pathways performed by Gene Set Variation Analysis (GSVA) in eight GEO-CRC cohorts. **Supplementary Table 6.** TME infiltration characteristics of samples in eight GEO-CRC cohorts. Table detailly lists the infiltration of 22 immune types of each sample which performed by CIBERSORT method in eight GEO-CRC cohorts. **Supplementary Table 7.** Difference of TME infiltration characteristics between Cluster_1 and Cluster_2 in Eight GEO-CRC Cohorts. The difference of TME infiltration characteristics between Cluster_1 and Cluster_2 in eight GEO-CRC cohorts. **Supplementary Table 8.** Difference of TME infiltration characteristics between WM_Score-high and WM_Score-low in eight GEO-CRC cohorts. The difference of TME infiltration characteristics between WM_Score-high and WM_Score-low in eight GEO-CRC cohorts. **Supplementary Table 9**. The correlation of writers and TME infiltration Characteristics. The Spearman correlation of gene expression of 26 writers and the infiltration of 22 immune cells in eight GEO-CRC cohorts. **Supplementary Table 10.** Differentially expressed genes between Cluster_1 and Cluters_2 associated with survival. The association of hypoxia status differentially expressed genes between Cluster_1 and Cluters_2, with patient overall survival times based on both univariate multivariate Cox proportional hazards models. **Supplementary Table 11.** Difference of miRNAs and miRNAs-DEGs target pathways between WM_Score-high and WM_Score-low in TCGA COAD/READ cohorts. The differentially expressed miRNA between WM_Score-high and WM_Score-low groups, and miRNA targeted mRNAs and mRNA enriched pathways. **Supplementary Table 12.** Difference of PDUI between WM_Score-high and WM_Score-low in TCGA COAD/READ cohorts**.** To compare PDUI between WM_Score-high and -low groups in TCGA COAD/READ cohorts, we used the t test and used Benjamini and Hochberg adjustment for FDR and considered an FDR < 0.05 and PDUI difference > 0.1 as statistical significance. **Supplementary Table 13.** The Univariate Cox regression analysis of PDUI and overall survival in TCGA COAD/READ cohorts. The association of PDUI for APA events with patient overall survival times Univariate Cox regression analysis in TCGA COAD/READ cohorts. **Supplementary Table 14.** Difference of A-to-I editing rate between WM_Score-high and WM_Score-low in TCGA COAD/READ cohorts. We used the t test to detect RNA editing sites with differential editing between WM_Score-high and -low groups in TCGA COAD/READ cohorts, and defined significantly differential editing sites as *p* < 0.05 and difference > 5%. **Supplementary Table 15**. The Univariate Cox regression analysis of A-to-I editing rate and overall survival in TCGA COAD/READ cohorts. The association of A-to-I editing rate with patient overall survival times Univariate Cox regression analysis in TCGA COAD/READ cohorts. **Supplementary Table 16.** The association of WM_Score and drug sensitivity in GDSC database. The Spearman correlation of WM_Score and drug sensitivity which quantified by AUC in GDSC database.

## Data Availability

The RNA sequencing or microarray data and clinical information of colorectal cancer patients, or patients with immune checkpoint were described in method section “Data collection and processing”. The resources, tools and codes used in our analyses were described in each method section in the methods.

## Abbreviations

3’UTR: 3′-untranslated region
A-I editing: Adenosine-to-inosine RNA editing
APA: Alternative polyadenylation
CMS: Consensus molecular subtypes
CRC: Colorectal cancer
DCs: Dendritic cells
DEGs: Differentially expressed genes
EMT: Epithelial-Mesenchymal Transition
FDR: False discovery rate
FPKM: Fragments Per Kilobase Million
GEO: Gene Expression Omnibus
GDSC: Genomics of Drug Sensitivity in Cancer
GSVA: Gene Set Variation Analysis
HR: Hazard ratio
ICB: Immune checkpoint blockade
miRNA: MicroRNA
PDUI: Poly A site usage index
CNV: Copy number variations
TAMs: Tumor-associated macrophages
TCGA: The Cancer Genome Atlas
Tfh cells: T follicular helper cells
TMB: Tumor mutation burden
TME: Tumor microenvironment
Tregs cells: T regulatory cells
WM_Score: “Writers” of RNA modification Score
