# Supplementary figures and images for: Cross-talk of four types of RNA modification writers defines tumor microenvironment and pharmacogenomic landscape in colorectal cancer
Source: Mol Cancer. 2021 Feb 8;20:29. doi: 10.1186/s12943-021-01322-w (PMC7869236; doi:10.1186/s12943-021-01322-w)

Figure S1

A

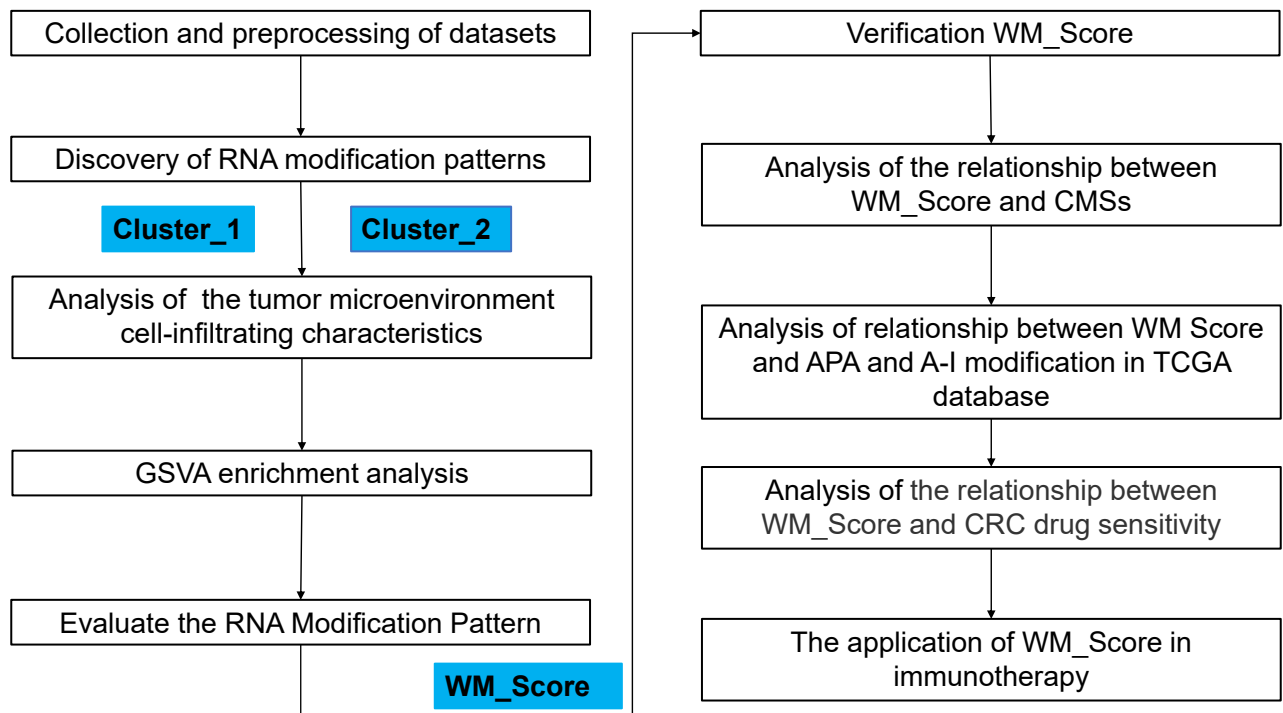

Figure S2

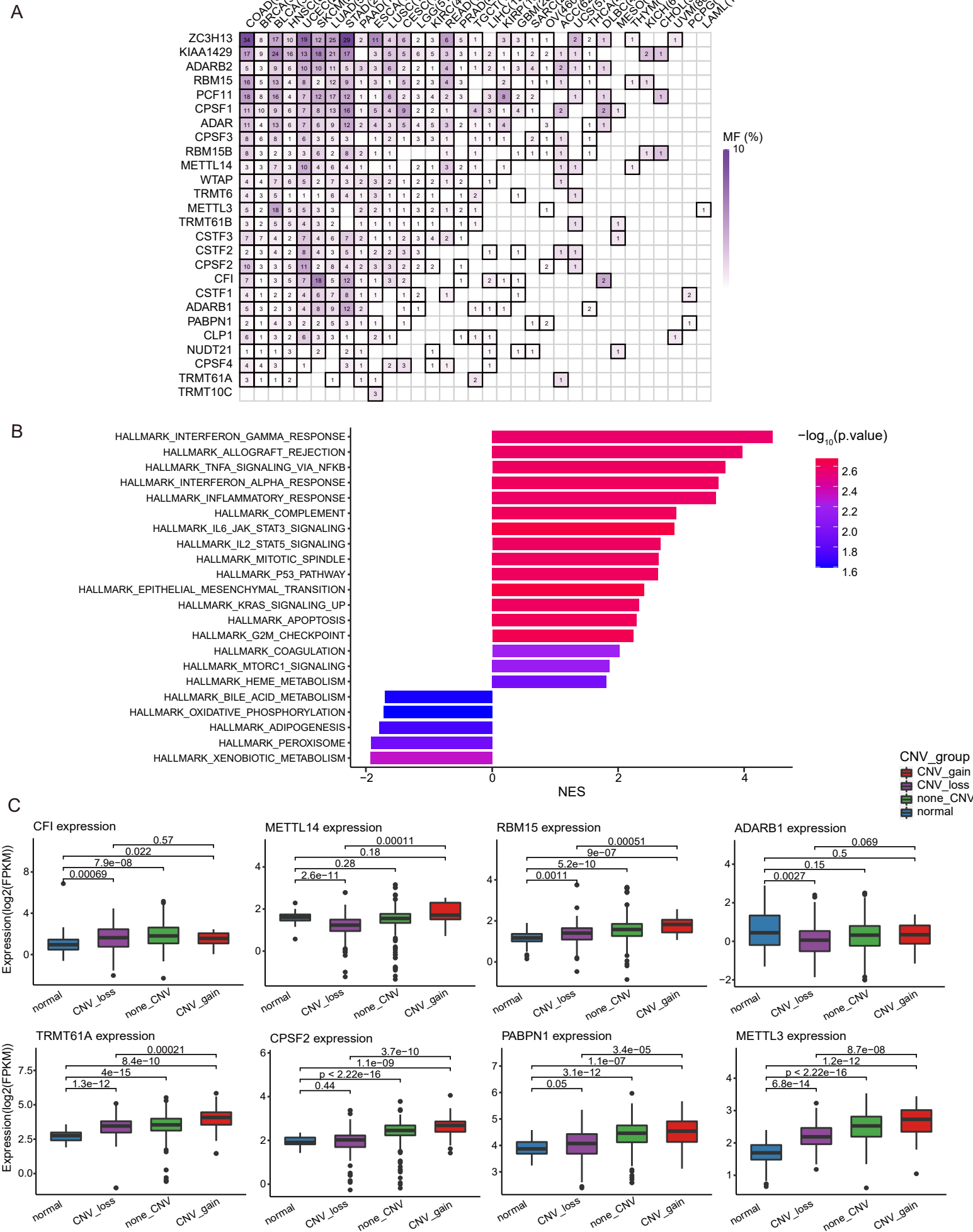

Figure S3

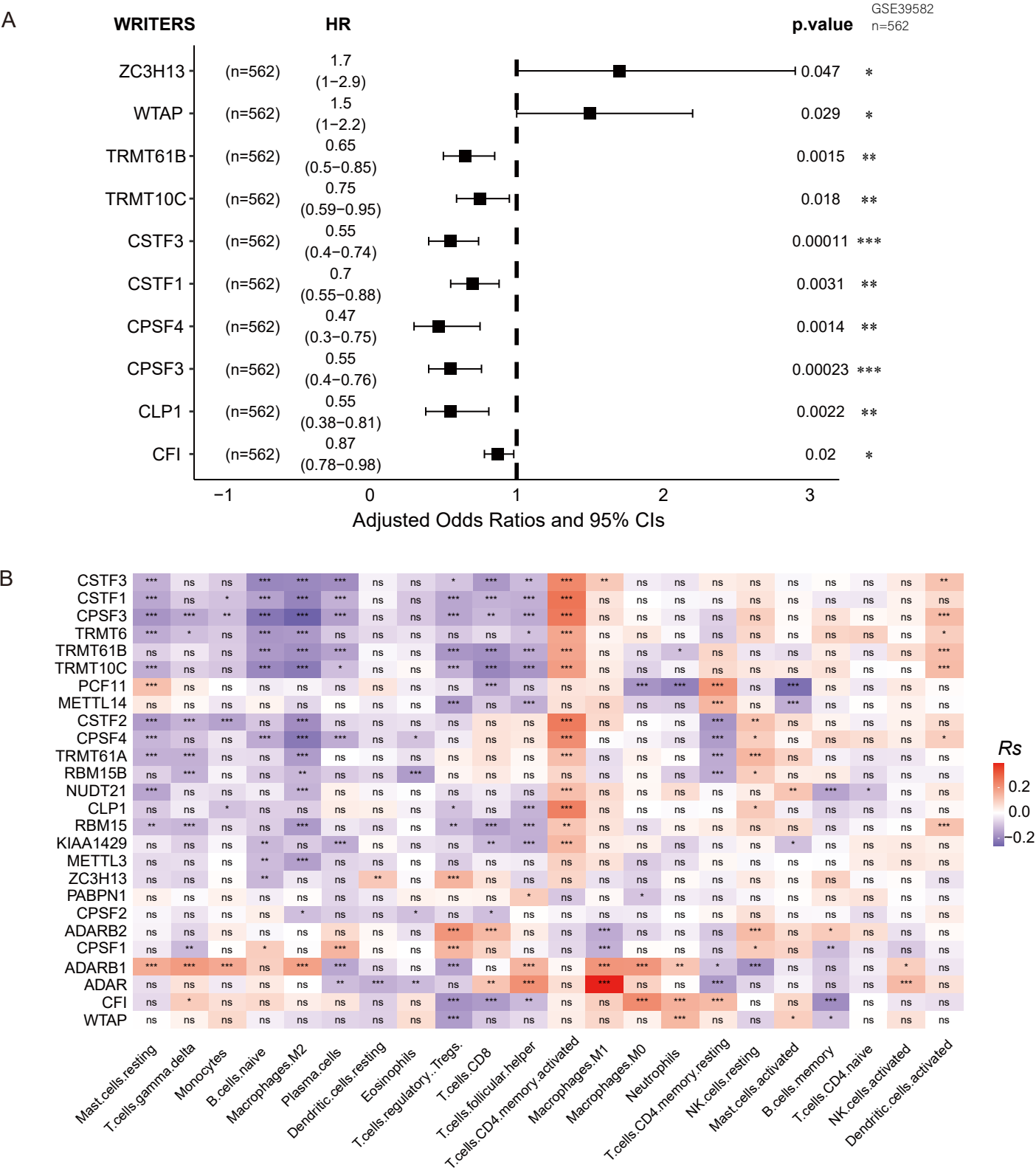

Figure S4

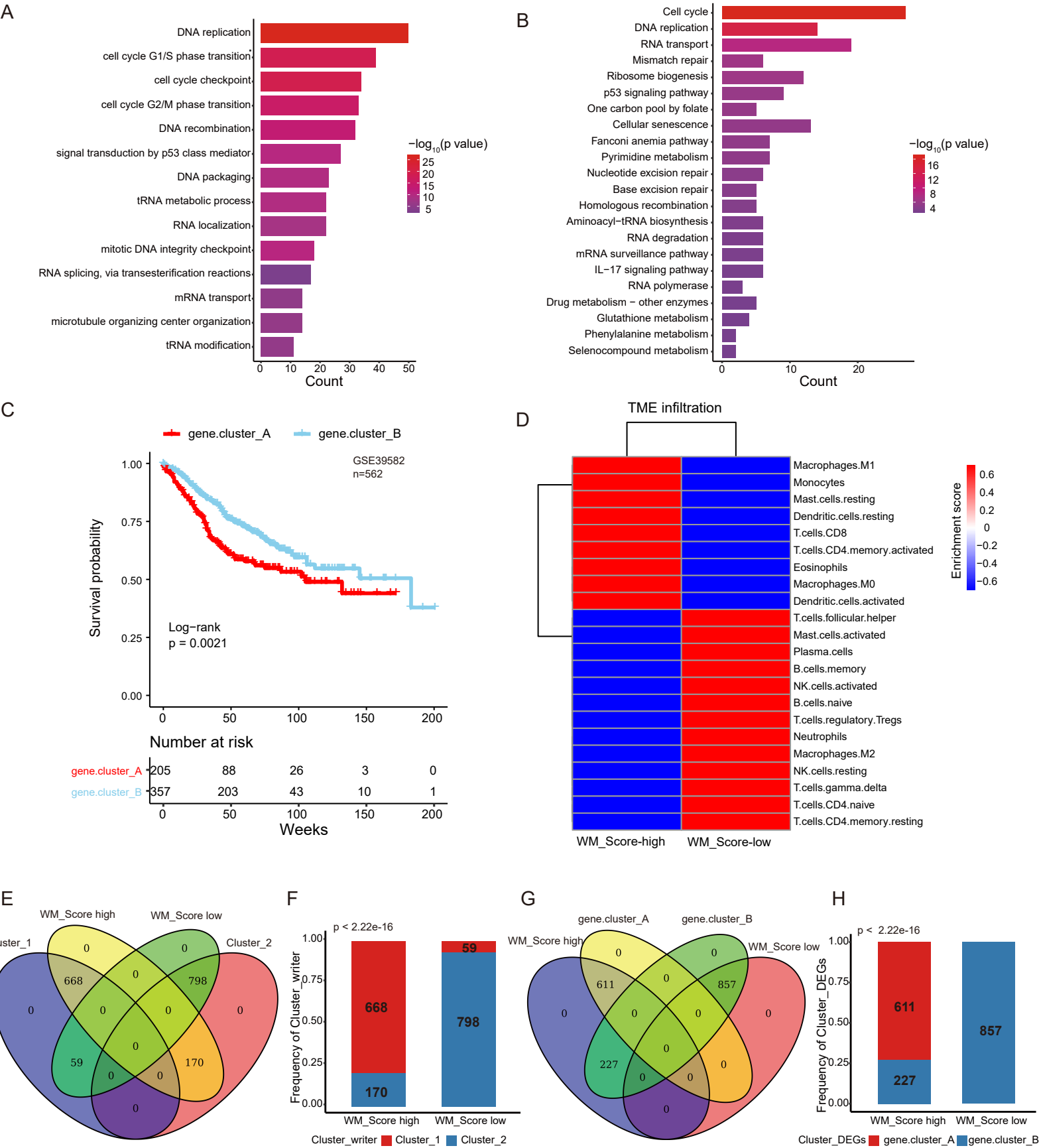

Figure S5

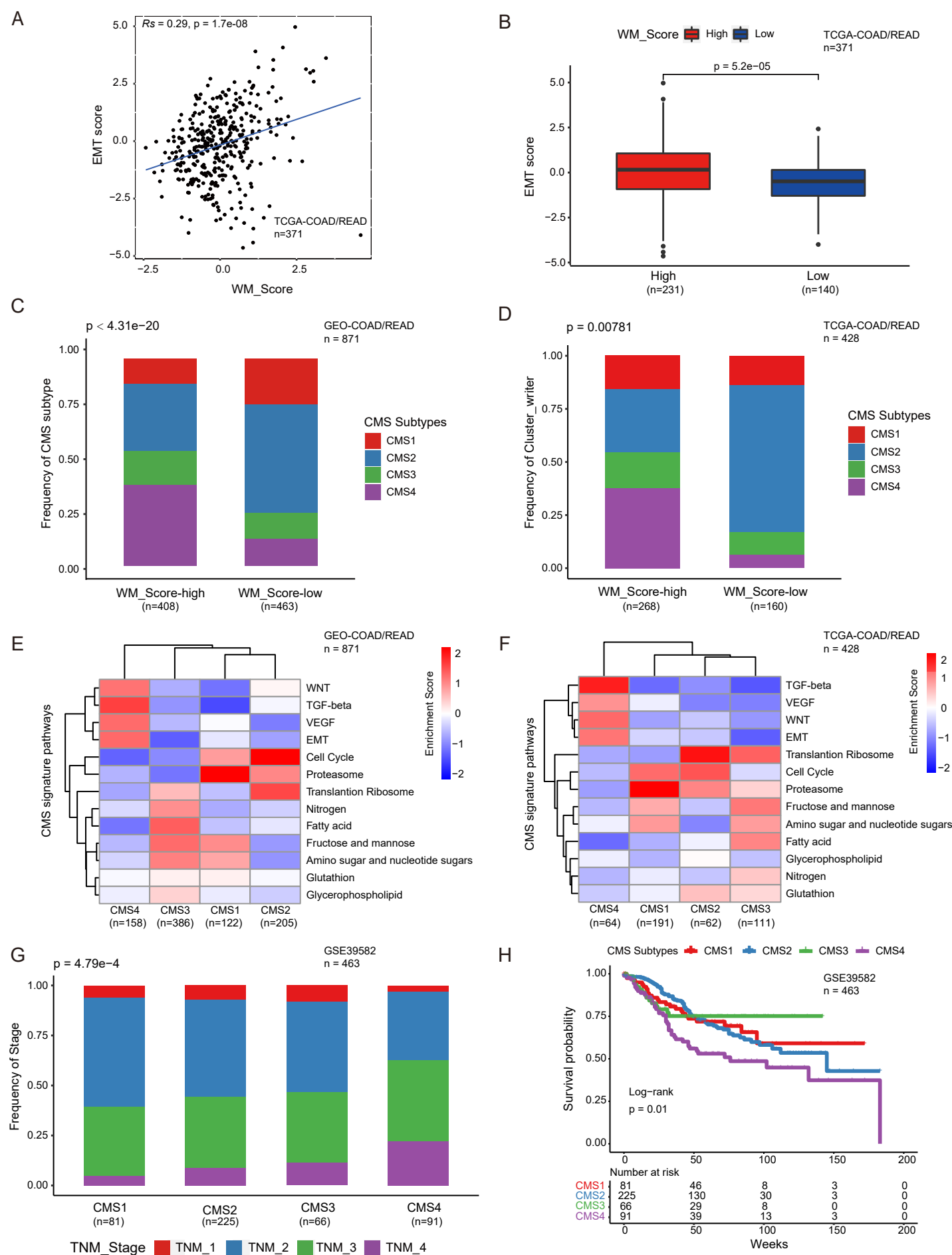

Figure S6

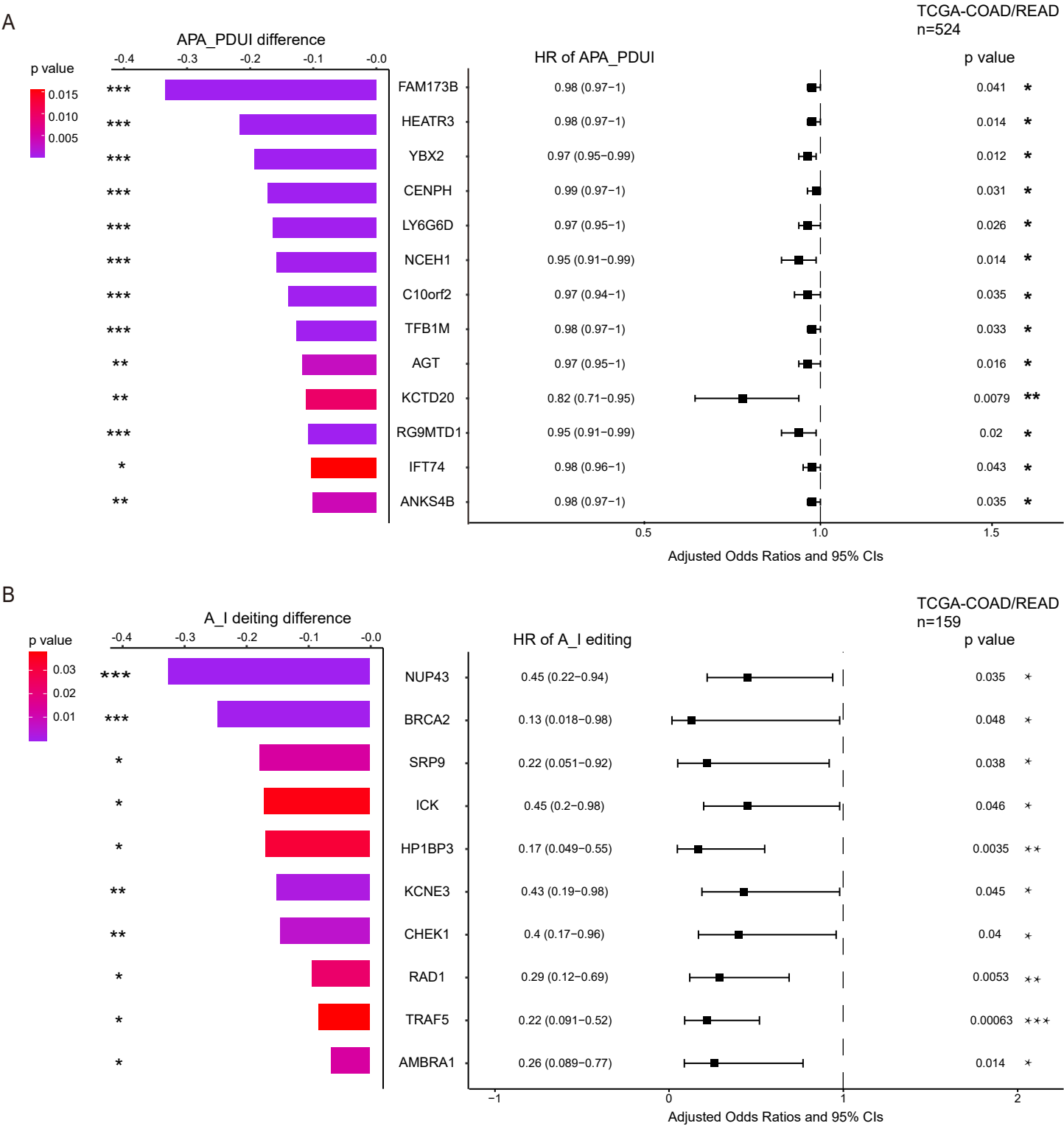

Figure S7

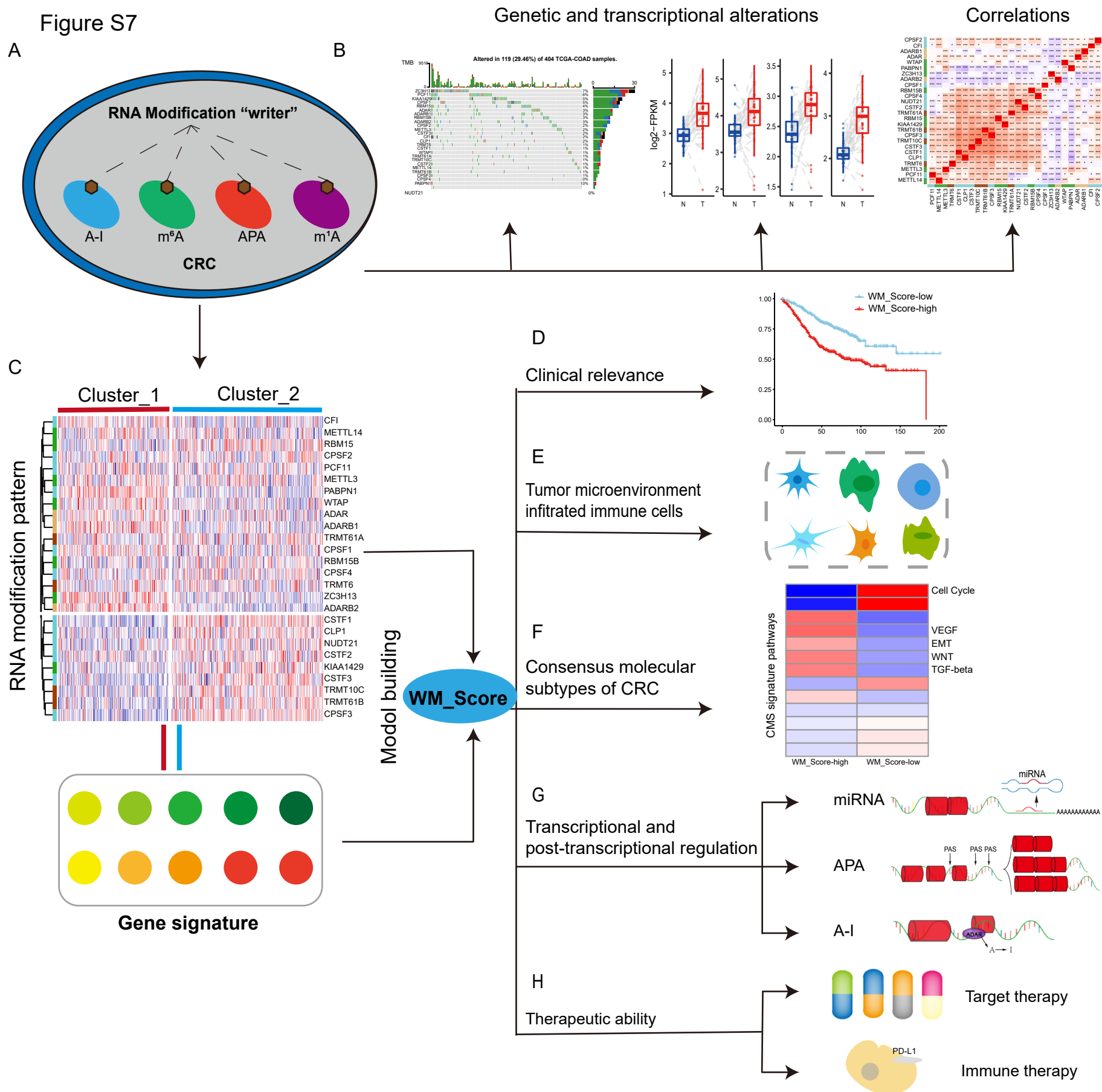

Supplement: Supplementary file 1 — Additional file 1 : Figure S1. Overview of study design. (A) Flowchart of the steps in the performed analyses. Figure S2. Analysis of mutation frequency and CNV in TCGA- COAD/READ. (A) The mutation frequency of RNA modification “writers” among 33 cancer types in the TCGA cohort. The horizontal axis represents cancer types, and the number of samples is given in the parentheses. The vertical axis lists the names of the genes. (B) Comparison of GSEA enrichment analysis between “writers” mutation samples and non-mutation samples. NES, Normalized enrichment score. (C) The distribution of correlation coefficient between “writers” expression and CNV in CRC. |Rs| > 0.3 and p value < 0.05 indicates that “writers” expression is related to CNV. (C) The expression of “writers” among CNV groups in CRC. The sample size for each group based on the CNV alteration (CFI, CNV_loss/ CNV_gain/ normal/ none_CNV = 138/18/54/305; METTL14, 143/19/54/299; RBM15: 125/17/54/319; ADARB1: 138/26/54/297; TRMT61A: 151/33/54/277; CPSF2: 155/33/54/273; PABPN1: 146/38/54/277; METTL3: 146/38/54/277). Wilcoxon test was used to assess the difference. The boxes indicate the median ± 1 quartile, with the whiskers extending from the hinge to the smallest or largest value within 1.5× IQR from the box boundaries. Figure S3. Biological characteristics of RNA modification “writers”. (A) Association of gene expression for 26 RNA modification “writers” with patient overall survival times based on Univariate Cox regression analysis in GSE39582 cohort. (B) Heatmap shows the positive (red) and the negative (blue) correlation between TME infiltration and WM_Score in CRC. *p < 0.05, **p < 0.01, and ***p < 0.001, as determined by the Spearman correlation analysis. Figure S4. Enrichment analysis of differentially expressed genes and the relationship between survival and the WM_Score. (A-B) GO (A) and KEGG (B) enrichment analysis of the 463 DEGs. The x-axis indicates gene counts within each GO term. The brightness of th [file 12943_2021_1322_MOESM1_ESM.pdf]
